# Genome-wide identification of wheat ABC1K gene family and functional dissection of *TaABC1K3* and *TaABC1K6* involved in drought tolerance

**DOI:** 10.3389/fpls.2022.991171

**Published:** 2022-08-29

**Authors:** Xiaoran Gao, Rong Zou, Haocheng Sun, Junxian Liu, Wenjing Duan, Yingkao Hu, Yueming Yan

**Affiliations:** College of Life Science, Capital Normal University, Beijing, China

**Keywords:** wheat, *ABC1K* genes, phylogenetics, *cis*-acting elements, drought stress, reactive oxygen species

## Abstract

Activity of BC1 complex kinase (ABC1K) serves as an atypical kinase family involved in plant stress resistance. This study identified 44 *ABC1K* genes in the wheat genome, which contained three clades (I–III). *TaABC1K* genes generally had similar structural features, but differences were present in motif and exon compositions from different clade members. More type II functional divergence sites were detected between clade I and clade III and no positive selection site were found in *TaABC1K* family. The three-dimensional structure prediction by Alphafold2 showed that TaABC1K proteins had more α-helixes with a relatively even distribution, and different clade members had differences in the content of secondary structures. The *cis*-acting element analysis showed that *TaABC1K* genes contained abundant *cis*-acting elements related to plant hormones and environmental stress response in the promoter region, and generally displayed a significantly upregulated expression under drought stress. In particular, both *TaABC1K3* and *TaABC1K6* genes from clade I was highly induced by drought stress, and their overexpression in yeast and *Arabidopsis* enhanced drought tolerance by suppressing active oxygen burst and reducing photosynthesis impairment. Meanwhile, *TaABC1K3* and *TaABC1K6* could, respectively, complement the function of *Arabidopsis abc1k3* and *abc1k6* mutants and reduce photosynthesis damage caused by drought stress.

## Introduction

As an evolved primitive atypical kinase family, activity of BC1 complex kinase (ABC1K) is widely distributed in prokaryotes and eukaryotes. The atypical protein kinases (aPKs) have little or no homology with eukaryotic protein kinases (ePKs), although their crystal structures have similar protein kinase folds (Bayer et al., 2012). ABC1Ks do not have many of the ePKs features as the functional domain HMM (Scheeff and Bourne, 2005), and generally possess the most conservative kinase motif, including VAIK catalytic motif, VAVK motif, and DFG motif (Jasinski et al., 2008). An ABC1K functional domain contains about 350 amino acid residues and 12 conserved motifs through analyzing the amino acid sequences of 100 ABC1K proteins from seven angiosperms (Lundquist et al., 2012a). Studies indicated that *ABC1K* proteins are localized in nucleoid, mitochondria, chloroplast, and plastoglobule of *Arabidopsis* (Gao et al., 2014), rice (Gao et al., 2011; Yang et al., 2012c), and maize (Gao et al., 2010). In *Arabidopsis*, 17 *ABC1K* family members were found, of which six were located in chloroplasts, mainly in plastids (Wang et al., 2011).

The first member of *ABC1K* family was found in *Saccharomyces cerevisiae*, which has the function of ensuring the correct formation of the cytochrome b6f complex by inhibiting the defects in cytochrome b mRNA translation produced by mutations in the *cbs2* gene of the nuclear translation activator (Bousquet et al., 1991; Bonnefoy et al., 1996). Since then, *ABC1Ks* have been found in rice (Yang et al., 2012c), maize (Gao et al., 2011), tomatoes (Li et al., 2015), and *Arabidopsis* (Jasinski et al., 2008). *AtABC1K8* (*AtOSA1*) was firstly identified in the chloroplast, which is essential for reactive oxygen species (ROS) scavenging under oxidative stress. The *AtABC1K8* mutant plants were more susceptible to cadmium toxicity, high light, and H_2_O_2_. In chloroplast, ABC1K could phosphorylate tocopherol cyclase VTE1 *in vitro* (Martinis et al., 2013, 2014), regulating vitamin E synthesis and recycling and promoting the production of α-tocopherol that function in the detoxification of ^1^O_2_ (Kruk and Trebst, 2008; Martinis et al., 2013, 2014). In various signaling pathways, protein phosphorylation catalyzed by protein kinase is an important mechanism in which plants respond to abiotic stress, as well as a major post-translational modification in the signaling pathway (Ichimura et al., 2000). The products of *ABC1K1* and its homolog *ABC1K3* play an important regulatory role in the metabolism of chlorophyll and photooxidative damage of plants (Yang et al., 2012a). *ABC1K1* and *ABC1K3* deletions in *Arabidopsis* caused significant decrease in chlorophyll content and the degradation of chlorophyll-binding proteins, and the structural components of quinones on the membrane system were also affected (Yang et al., 2012b; Lundquist et al., 2013).

Wheat (*Triticum aestivum* L., 2*n* = 6*x* = 42, AABBDD) is one of the three most important food crops with 17% of the world’s crop area as well as the main food source for 30% of the global population (Gill et al., 2004; Mayer et al., 2014). In natural conditions, extreme environmental stress such as salt, drought, and high temperature stress seriously affects wheat growth and development and causes a significant decline in grain yield and quality (Xiong et al., 2002). As the increasing world’s population as well as the great challenges for global food security, it is highly important to discover new stress-resistant genes and to improve the adaptability of wheat and other food crops.

TaABC1K proteins have more than 59.6% identities with the putative ABC1K proteins from *R. communis*, *P. tichocarpa*, and *Arabidopsis thaliana*. An aminoglycoside phosphotransferase choline kinase (APH_ChoK) domain is also present in the putative protein sequence of TaABC1K (Wang et al., 2011). The overexpressed wheat *TaABC1K* in *Arabidopsis* enhanced plant tolerance to drought, salt, cold stress, and stripe rust (Wang et al., 2013; Wu et al., 2020). Drought and rewatering experiments showed that 49–80% of the *TaABC1K* overexpressed plants survived whereas the corresponding survival rate in the wild type (WT) was only 25%. In addition, the maximum photochemistry efficiencies of PSII in the *TaABC1K* overexpressed plants under drought stress were significantly increased compared with WT. *TaABC1K* overexpressed plants had higher water retention and osmotic adjustment abilities, as well as decreased levels of damage to photosynthetic proteins and pigments, which were beneficial for enhancing tolerance to abiotic stresses. *TaABC1K* also serves as a regulatory factor participating in multiple stress-responsive genes such as DREB2A, RD29A, and ABF3 to decrease the damage of abiotic stresses (Wang et al., 2011). However, comprehensive studies on the structural and evolutionary characteristics and functional properties of the wheat *ABC1K* gene family still lack so far.

In this study, we used the newly released wheat genome database (IWGSC RefSeq v2.1) to perform a comprehensive genome-wide analysis of the wheat *ABC1K* gene family, and the function properties of *TaABC1K3* and *TaABC1K6* genes involved in drought response were further dissected through *S. cerevisiae* and *Arabidopsis* genetic transformation. We aim to further dissect the structure and molecular evolutionary characteristics of the wheat *ABC1K* gene family and its expression profiling and functional properties in response to abiotic stress.

## Materials and methods

### Retrieval and identification of wheat *ABC1K* genes

The amino acid sequences of 17 *AtABC1K* gene family members from *A. thaliana* were firstly downloaded from the Phytozome plant data website.^1^ The obtained AtABC1K protein sequences were used for BLASTP operation to retrieve the newly released wheat genome database (IWGSC RefSeq v2.1) in Ensembl Plants.^2^ The candidate TaABC1K sequences were analyzed through SMART (Letunic and Bork, 2018) and Pfam^3^ (Finn et al., 2014; Mistry et al., 2021) to verify if the obtained sequences contained conserved *ABC1* functional domain. HHMER was used to check the *TaABC1K* gene family based on the *ABC1* domain (Finn et al., 2011). The isoelectric point and molecular mass of wheat ABC1K proteins were identified using the ExPASy database^4^ and the results were analyzed by SPSS software.

### Chromosomal assignment and collinearity analysis of *TaABC1K* genes

The *TaABC1K* gene positions were determined by using *Ensembl Plant*s and MCScanX was used to analyze the amino acid sequences of *ABC1K* gene family members (Wang et al., 2012). The chromosomal localization and collinearity analysis of *ABC1K* gene family members were performed by TBtools (Chen et al., 2020).

### Phylogenetic and exon-intron structure analysis

Multiple sequence alignment for the identified ABC1K protein sequences was carried out by using MUSCLE software (Han et al., 2019).^5^ A Bayesian evolutionary tree was constructed by MrBayes 3.2.5 software according to the alignment files (Ronquist et al., 2012), and the reliability of the internal branches of the phylogenetic tree was evaluated through setting 1,000 cycles of self-priding resampling (Saitou and Nei, 1987; Tamura et al., 2013). The exon-intron structure characteristics of *ABC1K* gene family members were detected by gene structure display server (GSDS; Hu et al., 2014).^6^ The conserved motifs of ABC1K proteins were analyzed by the online MEME (Bailey and Elkan, 1994; Bailey et al., 2009).^7^ The maximum number of motifs was set to 15 and other parameters remained as default.

### Three-dimensional structure simulation by AlphaFold2 and molecular evolution analysis of ABC1K gene family

The three-dimensional (3D) structures of TaABC1K proteins from different clades were predicted using the online Alphafold2 website according to Duan et al. (2022). As the latest developed protein predicting tool, Alphafold has high accuracy and easy-to-use (Jumper et al., 2021). The type I and type II functional divergence sites in different subfamilies were detected using DIVERGE 3.0, and the critical value of posterior probability (Qk) was set to 0.8 (Gu et al., 2013). The site model in the PAML 4.4 software package (Yang, 2007) was used for positive selection analysis, using the site-specific model. When the non-synonymous substitution rate (dN) is higher than the synonymous rate (dS), in which the ratio ω (dN/dS) is higher than 1, it represents a positive selection. In this study, two pairs of models were selected using the BEB estimation method. The model comparison was according to Wu et al. (2014). Finally, the likelihood ratio test was used to detect the positive selections. Coevolution sites between amino acids were detected by using Protein Sequences (CAPS) in PERL software (Fares and McNally, 2006).

### The *cis*-acting element identification and RNA-seq expression analysis of *TaABC1K* genes

According to Li et al. (2021), 1,500 bp promoter region from the *TaABC1K* gene initiation codon was downloaded from the Ensembl Plant database, and the number and type of the *cis*-acting elements were analyzed by PlantCARE online website^8^ based on Rombauts et al. (1999). The publicly Chinese Spring (CS) wheat RNA-seq database was used to analyze RNA-seq data of TaABC1K encoding genes from the expVIP website.^9^ The transcriptional expression profiling at different grain developmental stages and in response to various stress treatments was detected. TPM was used for RNA-seq data and the data was normalized. TBtools was used for heatmap construction and cluster analysis according to Li et al. (2021).

### Plant materials, cultivation, and treatments

The seeds of wheat variety CS were sterilized and cultured based on the reported method by Han et al. (2019). The samples from seedling roots, stems, and leaves at the three-leaf stage and developing grains at 15 days post anthesis (DPA) were collected from three biological replicates. At the same time, seedlings at the three-leaf stage were treated with the following abiotic stress conditions: simulated drought [20% (W/V) PEG 6000] and oxidation stress (15 mM H_2_O_2_). Seedlings with normal culture condition were used as control (CK). The leave samples were collected after 12 h of treatment, and then all samples were quickly frozen with liquid nitrogen and stored in −80°C for later use. *Arabidopsis abc1k3* and *abc1k6* mutants were purchased from SALK website and *A. thaliana* Col-0 was used as WT. The *Arabidopsis* seeds were sterilized and cultured referred to Li et al. (2017).

### RT-qPCR

Total RNA extractions of wheat seedling leaves from different treatments and RT-qPCR were conducted according to Zhu et al. (2021). The internal reference gene was 18S, and CFX96 real-time PCR detection system (Bio-RAD, Hercules, CA, United States) was used to perform RT-qPCR. The relative gene expression levels were calculated by 2^−ΔΔCt^ method (Livak and Schmittgen, 2001; Yu et al., 2016). Three biological replicates were carried out on each sample and the significance of the experimental results was detected by one-way ANOVA.

### Subcellular localization

The subcellular localization of TaABC1K proteins was performed referring to the methods of Yoo et al. (2007) and Liu et al. (2018). GFP signal and chlorophyll red auto-fluorescence were observed and photographed by confocal laser scanning microscope (Zeiss LSM 780, Germany). The excitation light for GFP was 503–518 nm while the chloroplast spontaneous fluoresces were 590–608 nm.

### Overexpression of *TaABC1K* genes in *Saccharomyces cerevisiae*

The full CDS of *TaABC1K* genes were cloned into vector pYES2.0. Empty vector and recombinant vectors were transformed into yeast strain BY4741 referred to the standard procedures (Invirogen), and then cultured in SD-Ura (2% glucose) solid medium for 3 days. Single colonies were selected and cultured in liquid SD-Ura (2% glucose) medium until OD = 2.0, and then diluted to six stepped concentration and dropped onto a solid SD-Ura (2% glucose) medium with or without 5 mm H_2_O_2_ and 600 mM mannitol (simulated drought treatment).

### Overexpression and complementation of *TaABC1K* genes in *Arabidopsis*

The wild-type Col-0 and *abc1k3* and *abc1k6* mutants of *A. thaliana* were used as materials for overexpression and complementation experiments of *TaABC1K* genes, respectively. The recombinant plasmid pCAMBIA1302-*TaABC1K* was transferred into *Agrobacterium* GV3101 strain according to Weigel and Glazebrook (2006). Transgenic plants were generated using the stigma infiltration method (Bechtold and Bouchez, 1995) and consecutive identification and screening, in which the positive seedlings were selected on a ½ MS medium containing 50 mg/l kanamycin. Transgenic lines were generated after at least two generations of positive screening (Li et al., 2017).

### Determination of hydrogen peroxide and superoxide in *Arabidopsis* under drought stress

H_2_O_2_ and O_2_^−^ levels in *TaABC1K* overexpressed and complementary *A. thaliana* leaves were detected. The 5-day-old *Arabidopsis* seedlings were transferred into ½ MS petri dish and treated with 400 mM mannitol for 12 h. Seedlings in normal conditions were set as control group. H_2_O_2_ content was detected by DAB staining according to Wang et al. (2016) with minor modifications. The seedlings were soaked in 0.5% DAB with 10 mm Tris–HCl (pH 4) staining solution and vacuumed at 60 kPa for 10 min in dark, then chlorophyll was removed with bleaching solution (ethanol: acetic acid: glycerol = 3:1:1) in boiling bath. The O_2_^−^ level was measured by NBT staining, where the samples were soaked in 1 mM NBT with 200 mM K_2_HPHO_3_ (pH 6.2) and vacuumed at 60 kPa for 10 min in dark. The samples were immersed in fixative solution (10 ml methanol, 2 ml HCl, 38 ml H_2_O) for 15 min and decolorized with 7% NaOH and 60% ethanol in boiling bath. Seedlings were soaked in 50 mm DCFH-DA dye with 20 mM K_2_HPO_3_ and cultured in dark for 10 min. The roots of the treated seedlings were observed by confocal fluorescence microscope Zeiss LSM 780, and the excitation wavelength was set to 488.

### Measurement of chlorophyll content and chlorophyll fluorescence

Chlorophyll fluorescence was tested using an IMAGING PAM chlorophyll fluorescence meter (Walz, Effeltrich, Germany). *A. thaliana* plants were treated with or without drought stress for 1 week and preconditioned to complete darkness for more than 30 min before measurement (Schreiber et al., 2007). The experiment was conducted according to Zhu et al. (2021). The related photosynthetic parameters were obtained and calculated during the measurement, including the maximum photosynthetic efficiency by *F_v_*/*F_m_* = (*F_m_*-*F_o_*)/*F_m_*, and the photochemical efficiency of PSII in the light by Φ(PSII) = *F_v_^’^*/*F_m_^′^* = (*F_m_′*-*F_s_*)/*F_m_′* according to Genty and Baker (1989).

## Results

### Genome-wide identification of wheat *ABC1K* gene family

Firstly, 17 ABC1K protein sequences from *Arabidopsis* were used to perform BlastP in the Phytozome and Ensembl Plant websites that cover the latest genomic data of wheat. In total, 180 *ABC1K* gene family members from eight plant species were identified (Supplementary Table 1). In wheat, 44 *TaABC1K* genes were identified. The physicochemical properties of 44 TaABC1K proteins are shown in Supplementary Table 2, including gene ID, sequence length, molecular weight, isoelectric point, and the locations on chromosome. The results showed that the length of the coding sequence (CDS) of wheat *ABC1K* genes ranged from 1,425 to 3,373 bp encoding 373–954 amino acid residues. The molecular weight of ABC1K members ranged from 42 to 105 kDa, with an average of 72.31 kDa. The pI fluctuated between 5.01 and 9.97 (average value 7.67) with weak alkalinity. These results indicated that the members of the wheat ABC1K family had great differences in protein size, isoelectric point, and other physicochemical properties, suggesting their functional differentiation during the evolutionary process.

### Phylogenetic and structural analysis of *TaABC1K* genes

**Figure 1:**
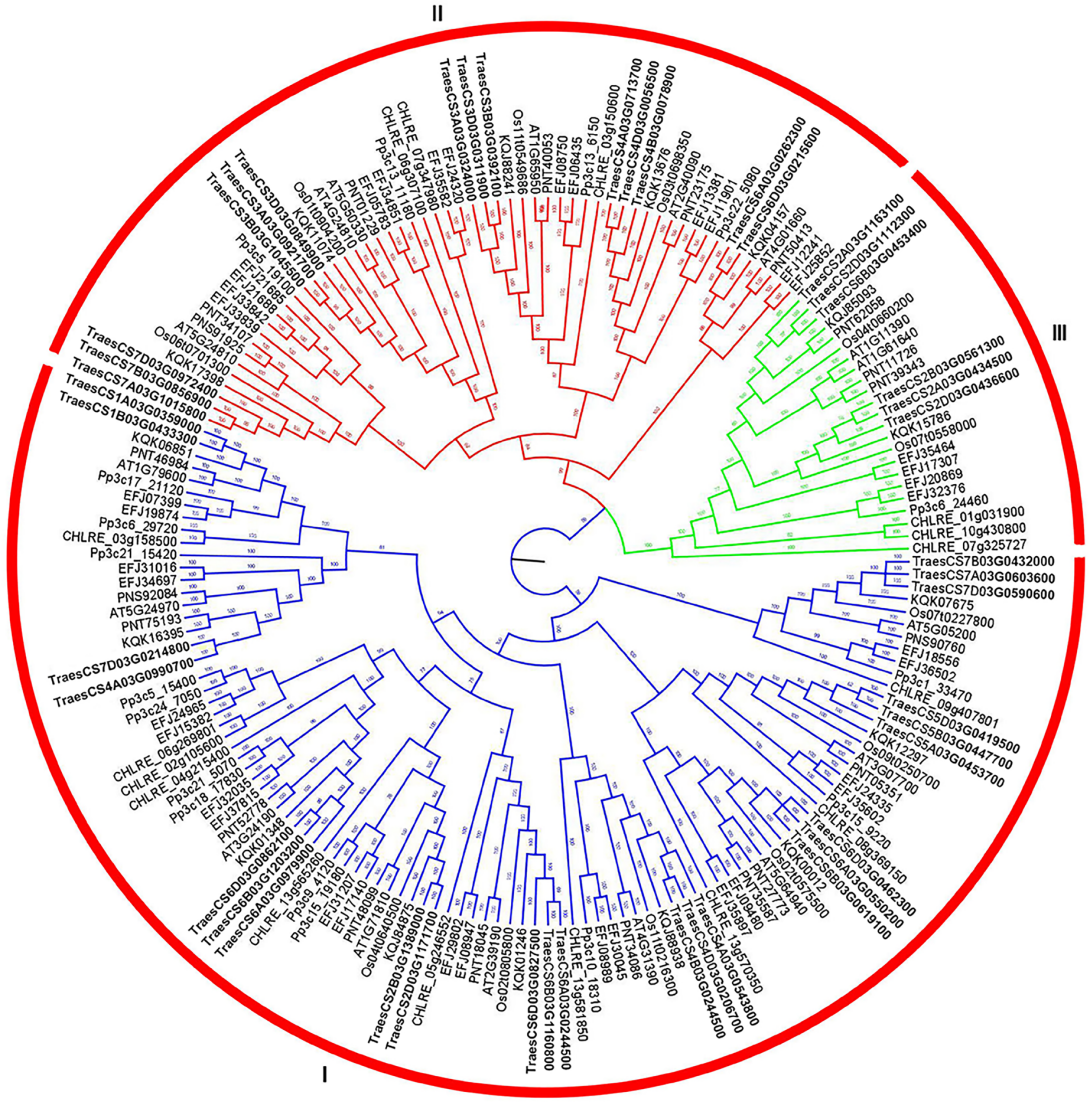
Bayesian phylogenetic tree of *ABC1K* gene family from *Triticum aestivum*, *Arabidopsis thaliana, Selaginella moellendorffii, Physcomitrella patens, Populus trichocarpa, Brachypodium distachyon, Chlamydomonas reinhardtii*, and *Oryza sativa*.

According to the Bayesian topology constructed by protein sequences from eight plant species (Figure 1), *ABC1K* gene family members were classified into three branches: Clade I (endoplast symbiotic branch), Clade II (mitochondrial endoplast symbiotic branch), and Clade III (ancestral branch). Most ABC1K genes belonged to clade I (101 genes), then clade II (56 genes), and clade III (23 genes). In particular, the *ABC1K* genes from each plant species were also classified into three same clades, indicating the close evolutionary relationships of plant *TaABC1K* gene family. Among 44 *TaABC1K* genes in wheat, 24, 14 and 6 members belonged to Clade I, Clade II, and Clade III, respectively (Supplementary Figure 1). An *ABC1* functional domain with about 120 amino acid residues was found in each *ABC1K* family member *via* SMART website. Additionally, *ABC1* functional domain had a VAVK-like motif (e.g., VAVK, VVIK, VAMK, or VVVK) and a DFG-like motif (e.g., DFG, DHG, or DVG; Supplementary Figure 2). All members included a VAVK-like motif except TraesCS4A03G0990700 and TraesCS2D03G1171700 in Clade I and a DFG-like motif except TraesCS6B03G0453400 in Clade III.

**Figure 2:**
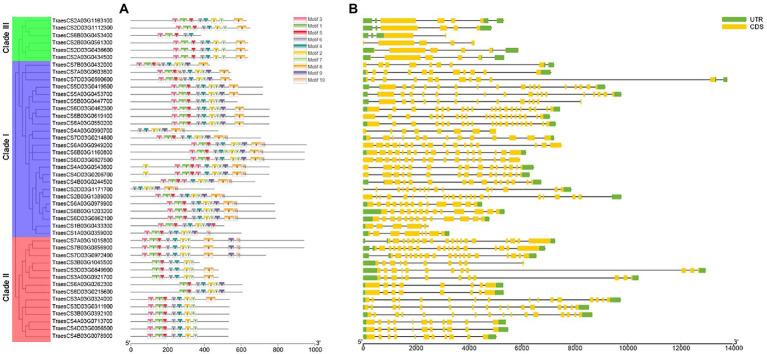
The motif and exon-intron structure of wheat ABC1K gene family members. **(A)** Conservative motifs of TaABC1K proteins. **(B)** Exon-intron structures of *TaABC1K* gene family.

The sequence motifs of each member of *TaABC1K* genes were analyzed by the online website MEME (Figure 2A). A total of 10 motifs were found, of which motifs 1–7 were present in 33 *TaABC1K* genes. In clade I, the number of motifs ranged from 7 to 11, and 19 in 24 genes had motif 1–7, in which 11 genes had 10 motifs. In clade II and clade III, the number of motifs ranged from 6–10 to 5–8, 9 in 14 members of clade II contained motif 1–7, and 5 in 6 members in clade III contained motif 1–8. According to the detection, the numbers and positions of motifs were relatively conserved among the internal members of each clade, but differences in the number of motifs were present in different clades.

The intron-exon structure characteristics of wheat *ABC1K* genes were analyzed by using the GSDS. The results demonstrated that the intron-exon compositions of *TaABC1K* genes from the same branch had similar structural characteristics but differed greatly among different branches (Figure 2B). In clade I, the number of exons ranged from 5 to 20, which fluctuated greatly compared to the other clades. Members of clade II had 8–18 exons with similar distributions while those in clade III only had 2–4 exons and 1–5 introns.

**Figure 3:**
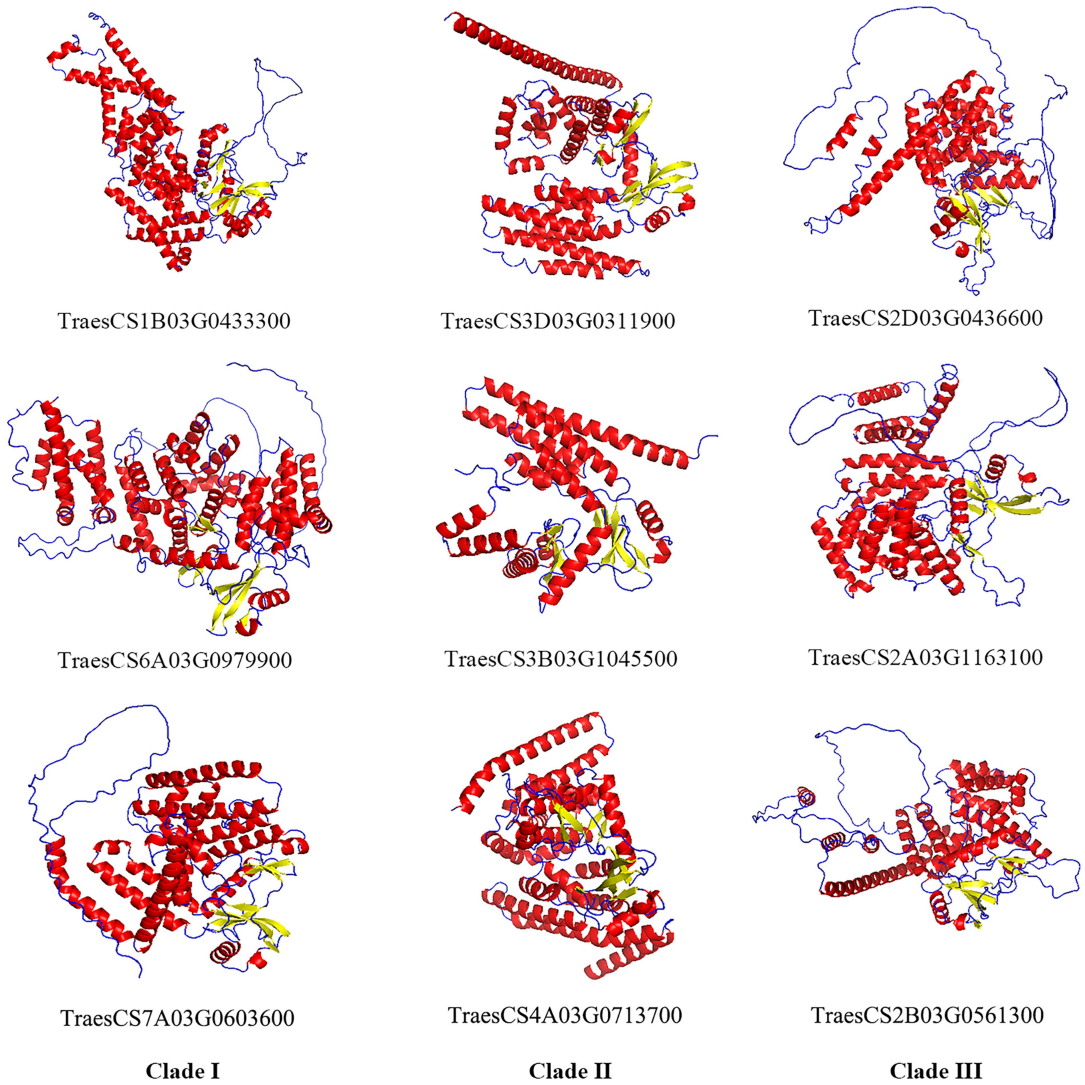
Three-dimensional structures of nine TaABC1K proteins from three different clades predicted by AlphaFold2. Red, α-helix; Blue, random coil; Yellow, β-sheet.

Alphafold2 was used to predict the 3D structures of nine TaABC1K proteins from different clades. Alphafold has shown highly successful predicting protein 3D structure from their amino acid sequences (Jumper et al., 2021), and has clear advantages such as its remarkable success in independent assessments of prediction accuracy, which can predict 98.5% of the human proteome (Ruff and Pappu, 2021) as well as multimer interactions (Mirdita et al., 2021). As shown in Figure 3, TaABC1K proteins were constructed mainly by α-helix and random coil, and generally had 11–18 α-helixes with a relatively even distribution. In particular, based on the results of three proteins selected from each clade, the calculation of the percentage of α-helix, random coil, and β-sheet showed that three TaABC1K proteins from clade II had significantly higher α-helix and β-sheet, which was 29 and 57% higher than those from clade III. The three proteins from clade III had significantly higher random coil while the content of α-helix, random coil, and β-sheet of three TaABC1K proteins from clade I were between clade II and clade III (Supplementary Table 3).

### Functional divergence, positive selection, and coevolution analysis of *TaABC1K* genes

The posterior probability (Qk) of divergence was set as Qk < 0.8 to screen important amino acid sites between every two clades according to Yang et al. (2005). As shown in Supplementary Table 4, the type I function divergence sites were found between clade I and clade III, and clade II and clade III with function divergence coefficient (θI) 0.28 to 0.5 and − 0.048 to 0.188, respectively. Four type I function divergence sites (331 V, 365Y, 336Q 331G) were identified between clade I and clade III, and one site (331 V) was identified between clade II and clade III. At the same time, 31 type II function divergence sites between clade I and clade III with θII 0.69 to 0.71 were found. In particular, the amino acid sites 331 V and 365Y belonged to both type I and type II functional divergence sites, suggesting that both sites underwent divergences concurrently. Two pairs of site models (M0/M3 and M7/M8) were used for positive selection analysis, and no positive selection sites were detected (Supplementary Table 5). Coevolution analysis identified 18 groups of coevolution sites (Supplementary Table 6), of which 9 groups were adjacent in the primary structure, and most of them showed different distributions from functional divergence sites. These coevolutionary sites might be beneficial for *TaABC1K*s to maintain the spatial structure and to adapt to environmental changes.

### Chromosomal assignment and collinearity analysis of *TaABC1K* genes

**Figure 4:**
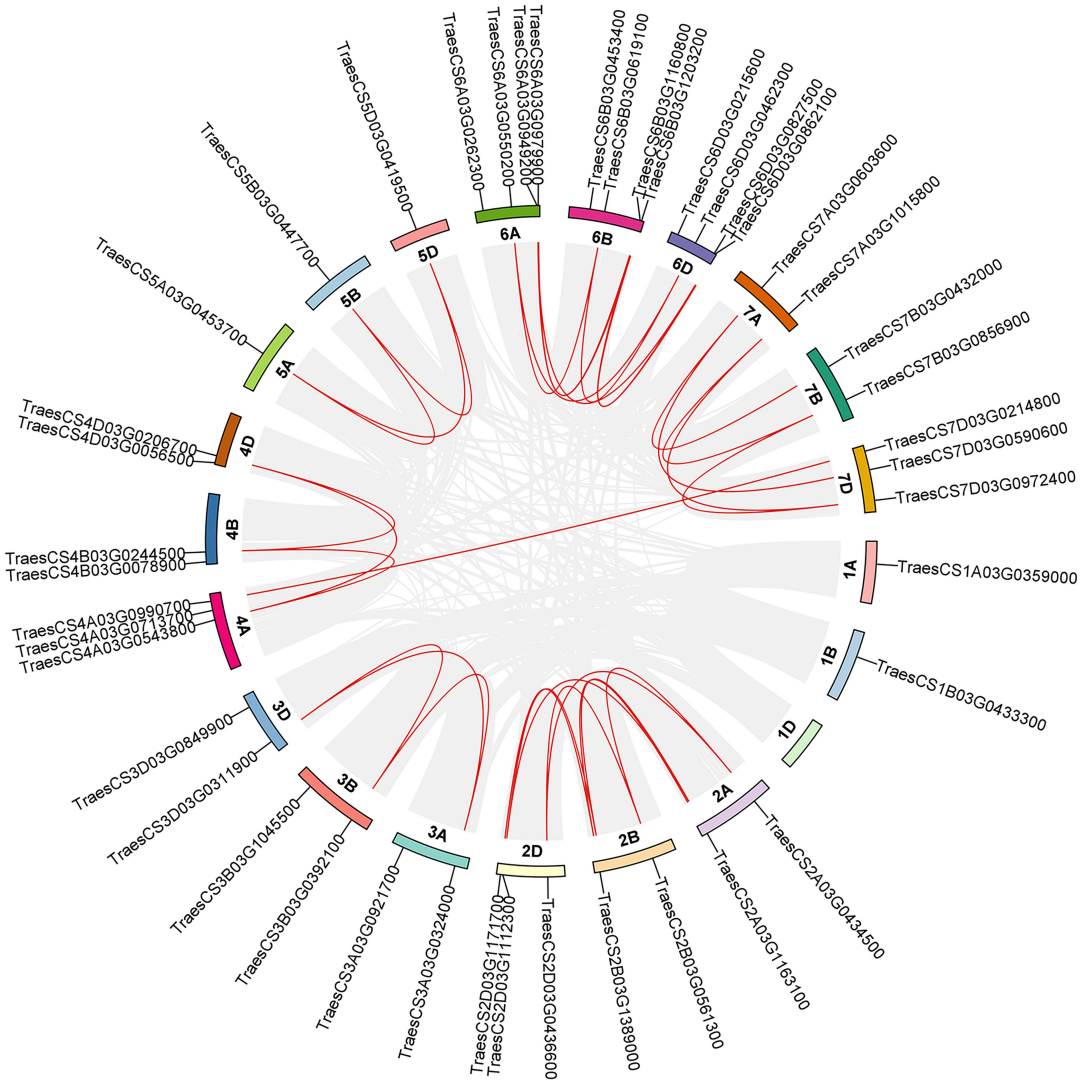
Distribution and collinearity of *ABC1K* gene family members in wheat chromosomes. The red lines represent the orthologous genes or tandem duplicated genes of *TaABC1K* and the gray lines represent the segmental duplication pairs in the whole wheat genome.

The distribution of 44 *TaABC1K* family members on chromosomes was analyzed referred to the latest CS wheat genome data. As shown in Figure 4, 44 *TaABC1K* genes were unevenly distributed on the 20 chromosomes of wheat with an even distribution on three subgenomes: 15 on the chromosome A, 14 on the chromosome B, and 15 on chromosome D. Collinearity analysis showed that most of the *TaABC1K* genes had orthologous genes on A/B/D chromosomes such as TraesCS3A03G0324000, TraesCS3B03G0392100, and TraesCS3D03G0311900 while most of the orthologous genes were clustered on the chromosome 6 and 7. However, the 1D chromosome had no *TaABC1K* genes that might lost during species evolution. In addition, TraesCS4A03G0990700 on chromosome 4A and TraesCS7D03G0214800 on chromosome 7D were paralogous genes, possibly caused by fragment repetition.

### Analysis of *cis*-acting elements in *TaABC1K* genes

The online tool PlantCARE was used to identify *cis*-acting elements in the 1,500 bp upstream promoter sequences of wheat *ABC1K* genes and the results are showed in Supplementary Figure 3. In general, *TaABC1K* genes from three branches had a similar composition of *cis*-acting elements (Supplementary Figure 3 A), also reflecting the evolutionary conservation of the *TaABC1K* gene family.

Light response elements were widely present in wheat *ABC1K* genes and 27 *cis*-acting elements related to light reaction were identified, including G-box, SP1, and Box4 (Supplementary Figure 3 B). Among them, G-box (CACGAC/CACGTG) was the most abundant light response element, and each subfamily member contained more than two copies of G-box elements on average. G-Box can combine with G-Box binding factors (GBFs) to regulate the expression of multiple genes (Gao et al., 2012). A total of 10 development-related elements were detected, of which CAT-box and CCGTCC-box were the most widely distributed. Each member contained one copy of each on average, suggesting that both elements in wheat *ABC1K* gene family play an important role in the regulation of plant growth and development.

The hormone response elements were also abundant in wheat *ABC1K* genes, 10 of which were detected. The most abundant elements were TGACG-motif and CGTCA-motif involved in the regulation of jasmonic acid synthesis as well as ABRE involved in response to abscisic acid. These three elements were present in almost every member of *TaABC1K* genes with an average copy number of 3.6, 3.1, and 4.2, respectively. Additionally, five environmental stress-related elements were detected in *TaABC1K* genes, including LTR, WUN-Motif, GC-motif, ARE, and TC-rich repeats. These elements were mainly involved in abiotic stress such as low temperature, hypoxia, drought, and cold stresses. Among them, LTR, GC-Motif, and ARE were the most widely distributed (Supplementary Figure 3 B), which responded to low temperature, hypoxia, and anaerobic stress, respectively.

Two *TaABC1K* genes TraesCS1B03G0433300 from chromosome 1B (named as *TaABC1K3*) and TraesCS6A03G0979900 from chromosome 6A (named as *TaABC1K6*) were selected for further analysis, which were from clade I and had a similarity of 74 and 77% to *Arabidopsis ABC1K3* and *ABC1K6*, respectively. *TaABC1K3* had TCT-motif and G-box involved in light responsiveness, MBS (MYB binding site) involved in drought-inducibility, TC-rich repeats involved in defense and stress response as well as ABRE involved in abscisic acid response. *TaABC1K6* contained GATT-motif and GC-motif involved in light and anoxic response as well as ABRE, MBS, and G-box. Thus, the *cis*-elements of *TaABC1K3* and *TaABC1K6* mainly participated in light and stress responses.

### Transcription expression profiling of *TaABC1K* genes in different organs and in response to abiotic stresses by RNA-seq

The publicly available RNA-seq data of 44 *TaABC1K* genes were obtained, and heat maps were constructed (Supplementary Figure 4). The expression profiling in different organs (Supplementary Figure 4 A) showed that most *TaABC1K* genes had a high expression level in leaves and shoots, and some genes had medium expression level in roots, but the expression level in the developing grains was relatively low. In particular, *TaABC1K* genes from clade I generally had a high expression in leaves and shoots such as *TaABC1K3* and *TaABC1K6* whereas most of the genes from clades II and III showed a relatively low expression level in different organs.

*TaABC1K* genes generally displayed an upregulated expression under different abiotic stresses (Supplementary Figure 4 B). Most of the genes from clade I were upregulated under multiple abiotic stresses, especially under drought and 2-week cold stresses, suggesting the potential roles of *TaABC1K* genes in clade I in response to abiotic stresses. 11 in 13 genes in clade I were upregulated under drought or PEG stress with a maximum increase of 74%. In particular, *TaABC1K3* had a significantly upregulated expression under heat (+38%), drought or PEG (+32%), and cold stresses (+49%). In contrast, the expression levels of the genes in clade II and clade III were relatively low in wheat. Although some of the data were missing, about half of the genes were still upregulated under PEG and cold stresses. Notably, the genes in clade I upregulated by up to 20 and 74% under drought and PEG stress, respectively, significantly higher than those from clade II (−3 and 35%, respectively), but lower than those of clade III (93 and 102%, respectively), possibly caused by their functional differentiation during the evolutionary process.

### Subcellular localization of *TaABC1K3* and *TaABC1K6* and their expression patterns in different organs and abiotic stresses by RT-qPCR

**Figure 5:**
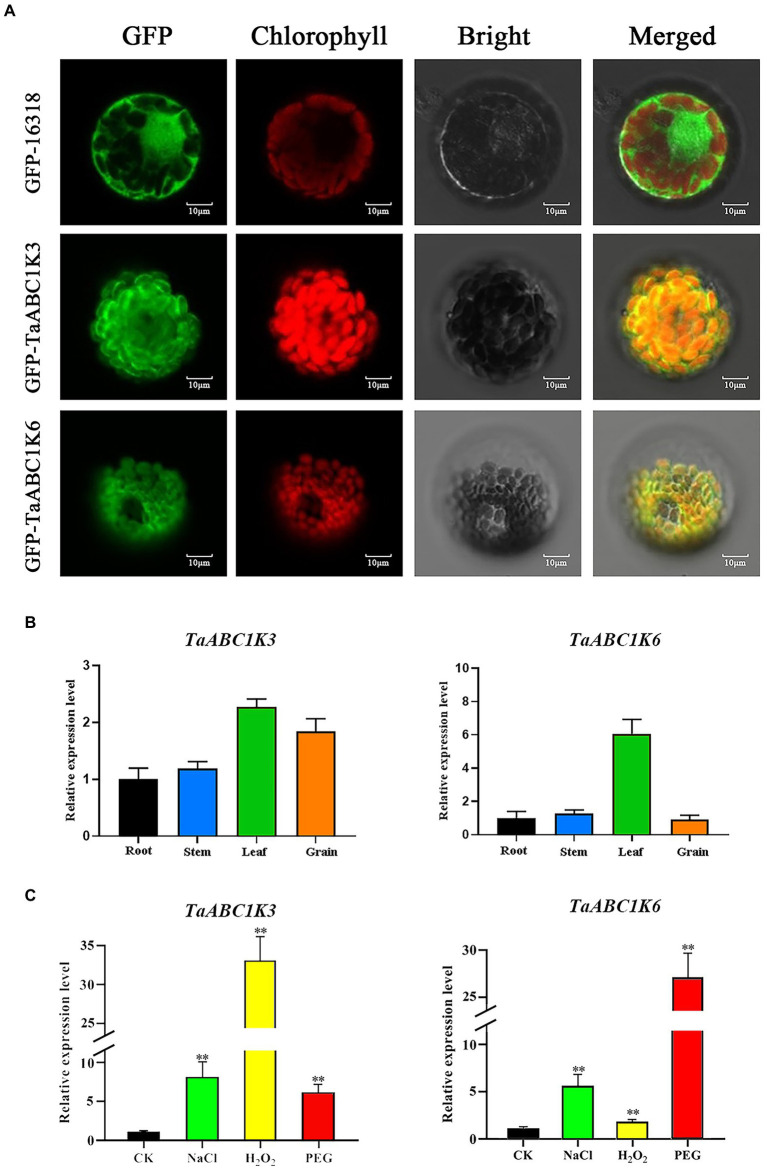
Subcellular localization and RT-qPCR analysis of *TaABC1K3* and *TaABC1K6*. **(A)** Subcellular localization of TaABC1K3 and TaABC1K6. GFP was induced fluorescence, chlorophyll is chloroplast autofluorescence. **(B)** RT-qPCR analysis of *TaABC1K3* and *TaABC1K6* genes in wheat different tissues. **(C)** RT-qPCR analysis of *TaABC1K3* and *TaABC1K6* genes under abiotic stresses. Statistically significant differences between control group and treatment group were calculated by an independent Student’s *t*-tests: **p < 0.01.

The subcellular localization results of TaABC1K3 and TaABC1K6 observed by confocal scanning laser microscopy indicated that the GFP fluorescence of both TaABC1K3 and TaABC1K6 proteins was present in chloroplasts (Figure 5A). This confirmed that both proteins were localized in the chloroplast.

The transcription expression patterns of *TaABC1K3* and *TaABC1K6* genes in roots, stems, leaves, and developing grains of CS were further detected by RT-qPCR. The specific primers were designed and the primer sequences are shown in Supplementary Table 7. The results showed that the highest expression level of both *TaABC1K3* and *TaABC1K6* genes occurred in leaves. Meanwhile, *TaABC1K3* also had a high expression in the developing grains while *TaABC1K6* displayed a lower expression in roots, stems, and developing grains (Figure 5B). When subjected to PEG, NaCl and H_2_O_2_ stresses, both *TaABC1K3* and *TaABC1K6* genes were significantly upregulated (Figure 5C). Particularly, the expression levels of *TaABC1K3* and *TaABC1K6* under PEG drought stress was dramatically increased by 6 and 27 times compared to the control, respectively. In addition, *TaABC1K3* also showed a sharp upregulation of 33 times under H_2_O_2_ stress, which all showed statistically significant compared with the control group.

### Overexpression of *TaABC1K3* and *TaABC1K6* in yeast and *Arabidopsis* enhanced drought tolerance

**Figure 6:**
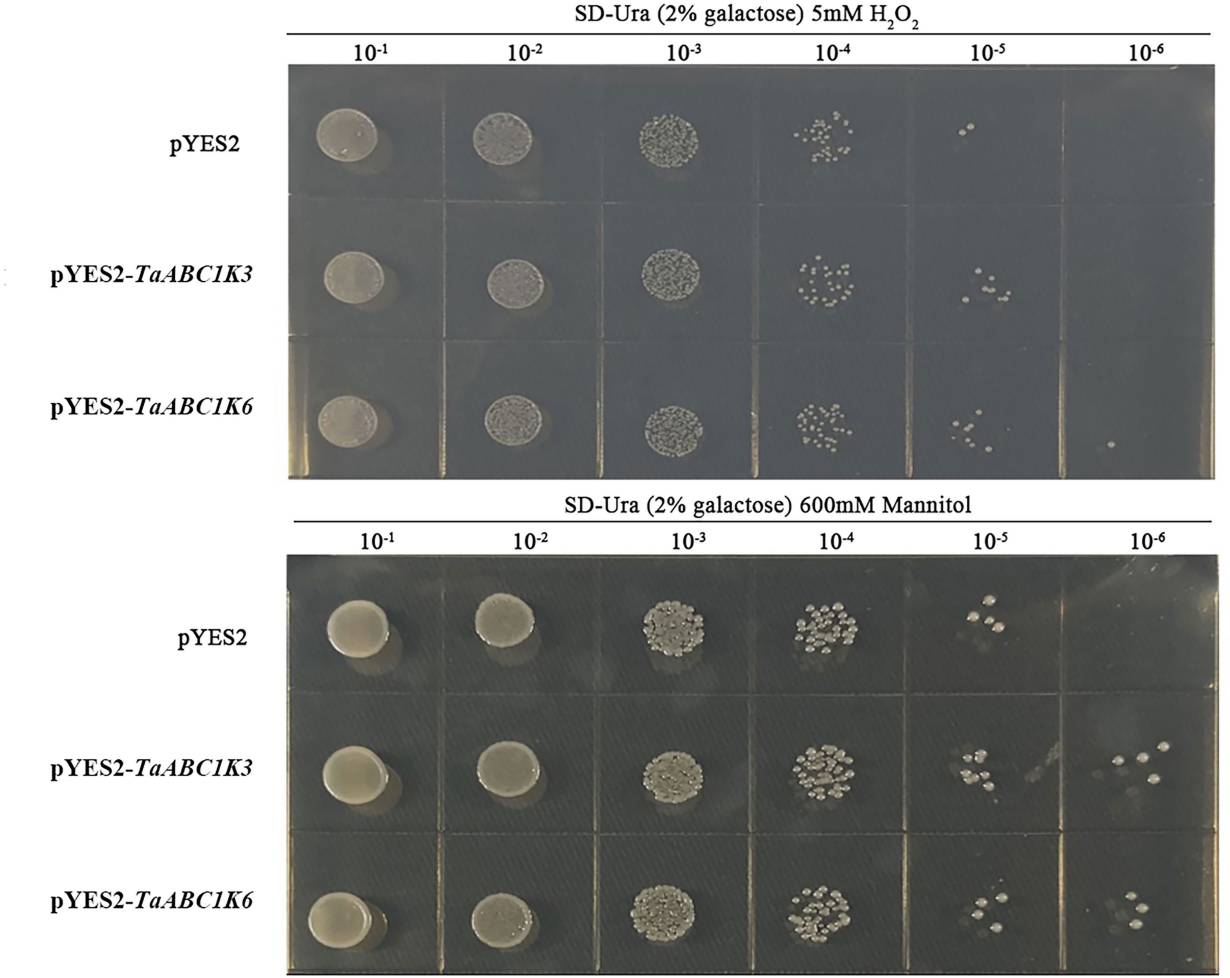
Overexpression of *TaABC1K3* and *TaABC1K6* genes enhanced the tolerance of yeast to abiotic stress.

Yeast transformation experiment showed that the growth rate of wild-type and *TaABC1K3 and TaABC1K6* overexpressed yeast strains under drought and H_2_O_2_ stresses were inhibited to different degrees along with the dilution of yeast culture medium concentration. However, the inhibition of *TaABC1K3* and *TaABC1K6* overexpressed yeast strains was significantly lower than that of the no-load strain (Figure 6). These results indicated that *TaABC1K3* and *TaABC1K6* genes could enhance the tolerance of yeast to drought and H_2_O_2_ stresses.

Further genetic transformation experiments of *TaABC1K3* and *TaABC1K6* genes to *Arabidopsis* wild-type Col-0, *abc1k3*, and *abc1k6* mutants were conducted. Six homozygous single-copy transgenic lines were produced through consecutive identification and screening, named OE3-1, OE3-2, and OE3-3 for *TaABC1K3* and OE6-1, OE6-2, and OE6-3 for *TaABC1K6*. Two complementary lines were generated, named *abc1k3/6* 35S: *TaABC1K3/6*. Inflorescence staining showed that *TaABC1K3* and *TaABC1K6* genes were integrated into the chromosomes of *Arabidopsis*. PCR identification confirmed that *TaABC1K3* and *TaABC1K6* genes were successfully transferred into overexpressed and complementary *Arabidopsis* plants (Supplementary Figure 5).

**Figure 7:**
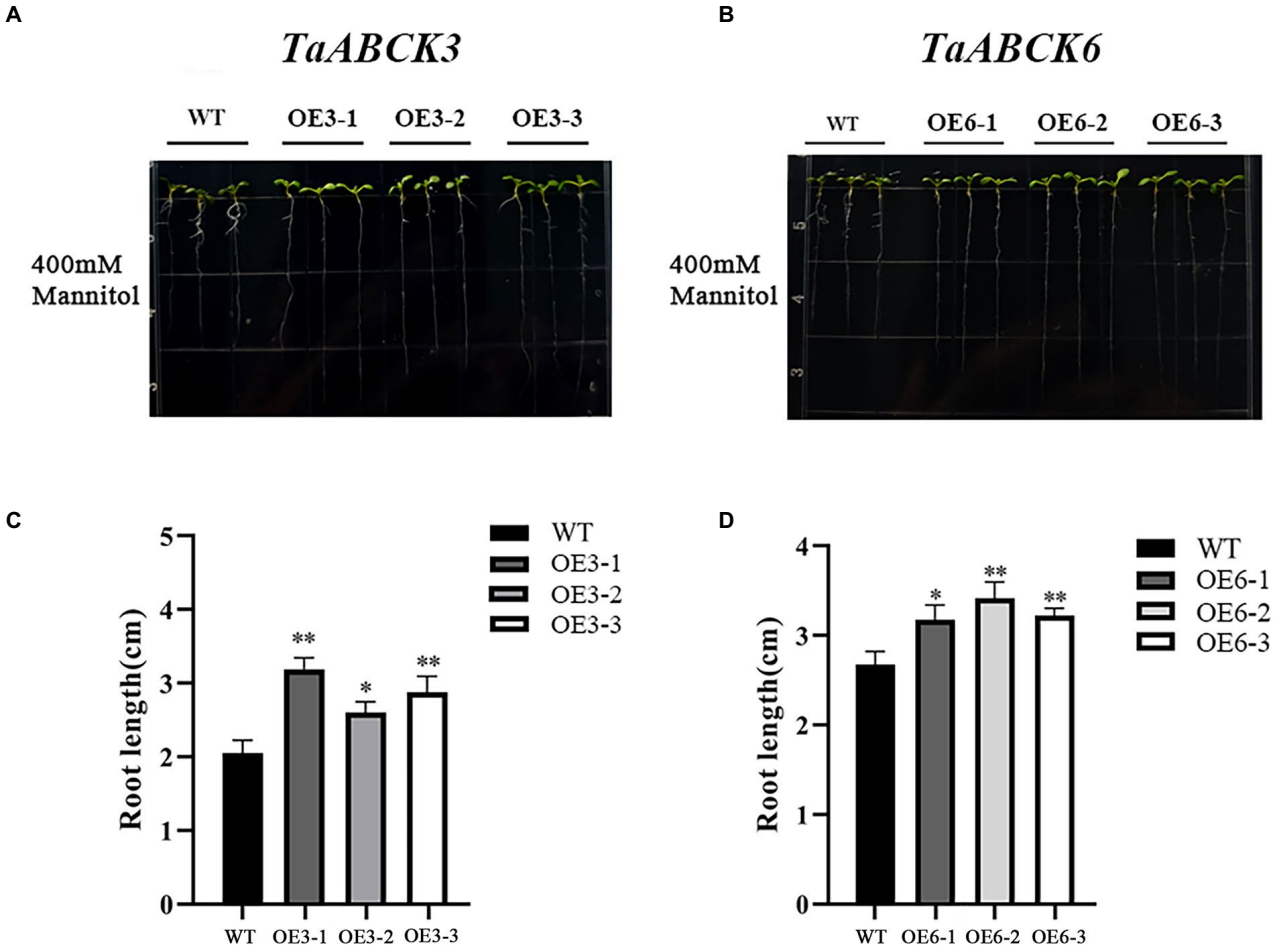
Overexpressed of *TaABC1K3* and *TaABC1K6* enhanced the drought tolerance of *Arabidopsis* seedlings. Phenotypic changes in wild-type **(A)** and *TaABC1K3* and *TaABC1K6* overexpressed seedlings **(B)**, relative fresh weight of overexpressed *Arabidopsis* **(C)**, and wild type **(D)** are indicated. Mean ± SD of data from three independent biological replicates. Statistically significant differences between wild type and overexpressed plants were calculated by an independent Student’s *t*-tests: *p < 0.05; **p < 0.01.

**Figure 8:**
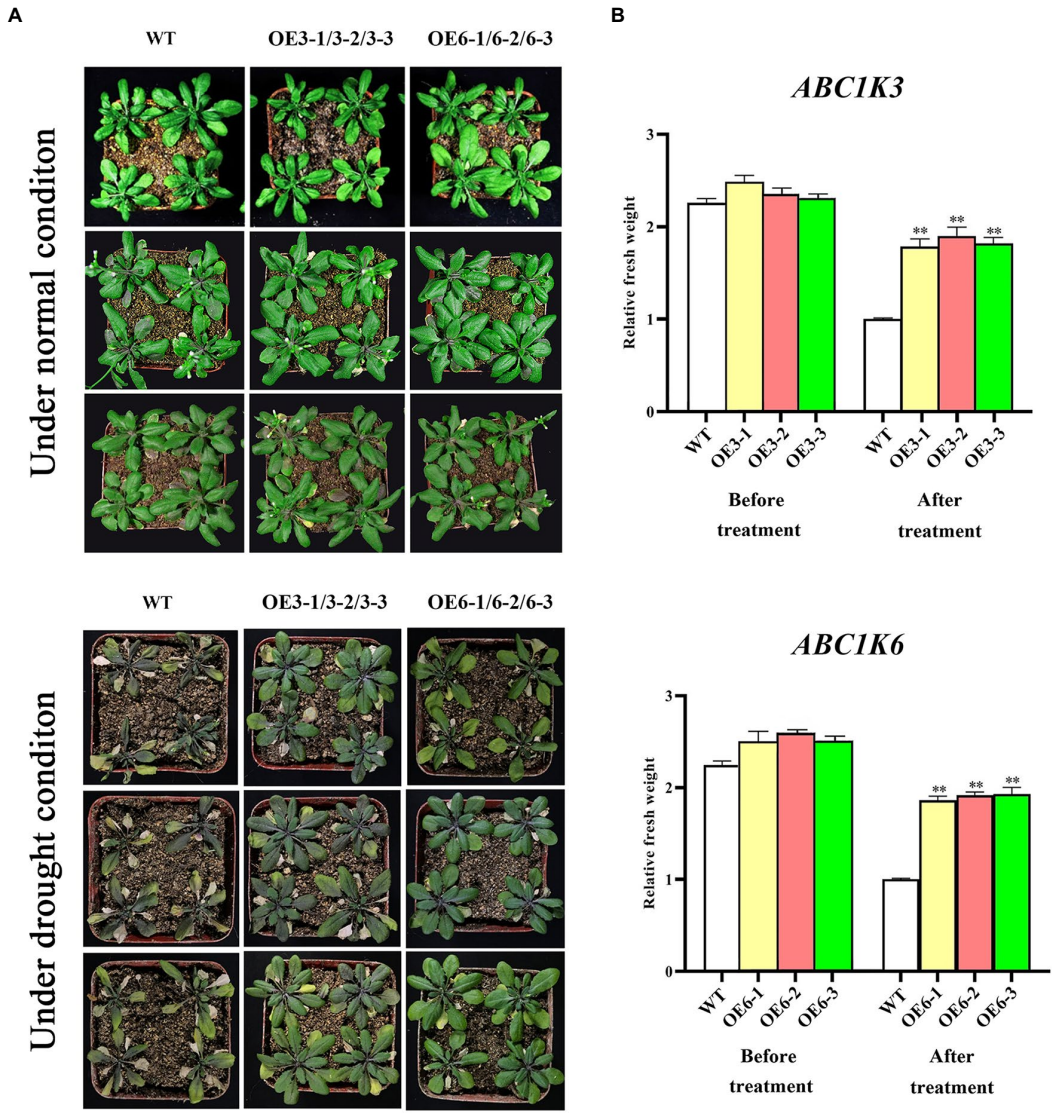
Phenotype changes of *TaABC1K3* and *TaABC1K6* overexpressed *Arabidopsis* plants under soil culture in response to drought stress. **(A)** Soil drought stress treatment was performed on wild-type, *TaABC1K3* and *TaABC1K6* overexpressed plants. **(B)** Comparison of fresh weight of wild-type, *TaABC1K3*, and *TaABC1K6* overexpressed plants under drought treatment and normal conditions. Statistically significant differences between control group and treatment group were calculated by an independent Student’s *t*-tests: **p < 0.01.

Phenotypic changes of *TaABC1K3* and *TaABC1K6* overexpressed plants and WT under drought stress are shown in Figures 7A,B. Under 400 mM mannitol simulated drought stress, the inhibition degree of root length in the *TaABC1K3* and *TaABC1K6* overexpressed plants was significantly lower than that of wild-type plants (Figures 7C,D), implying that both *TaABC1K3* and *TaABC1K6* genes could improve the resistance of *Arabidopsis* to drought stress. Further soil culture of transgenic lines under drought stress indicated that the wild-type plants had obvious chlorosis in leaves (Figure 8A) and the fresh weight was significantly reduced (Figure 8B). On the contrary, leaf chlorosis of *TaABC1K3* and *TaABC1K6* overexpressed plants was significantly mitigated, and the fresh weight was not declined significantly compared with the WT, demonstrating that the overexpressed *TaABC1K3* and *TaABC1K6* plants had better resistance to drought stress.

### *TaABC1K3* and *TaABC1K6* suppressed active oxygen burst and reduced photosynthesis impairment triggered by drought stress

**Figure 9:**
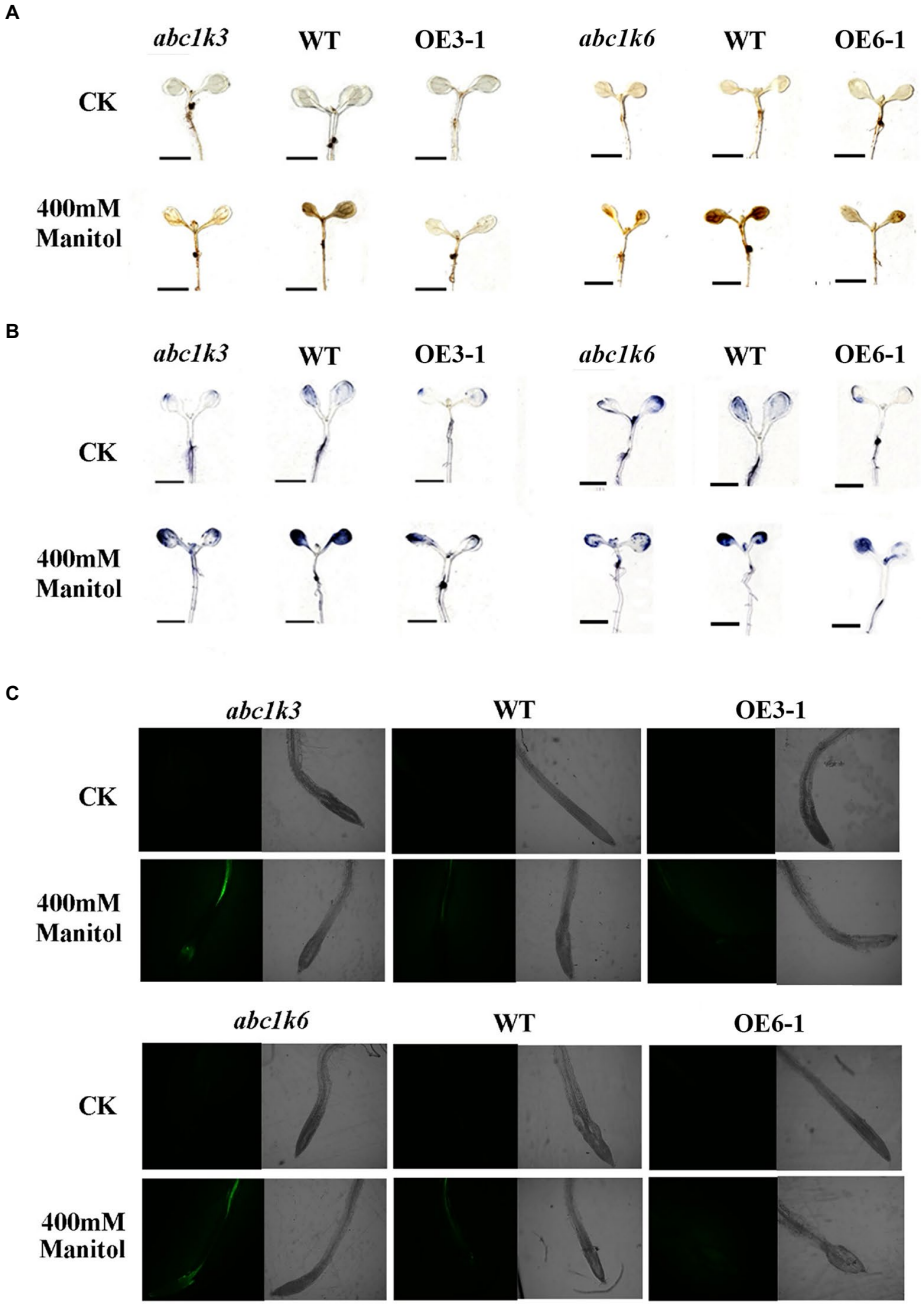
Overexpression of *TaABC1K3* and *TaABC1K6* suppressed the active oxygen burst triggered by drought stress in *Arabidopsis* mutant *abc1k3* and *abc1k 6*. **(A,B)** Differences of ROS content in *abc1k3/6* mutant, wild-type and *TaABC1K3/6* overexpressed plants under 400 mM mannitol stress. The seedlings were stained by diaminobenzidine (DAB; **A**) to form brown precipitates in the presence of hydrogen peroxide, or by nitroblue tetrazolium staining (NBT; **B**) to produce blue precipitate in the presence of superoxide (Scale bar =5 mm). **(C)** Seedlings of *abc1k3/6* mutant, wild-type and *TaABC1K3/6* overexpressed plants were subjected to DCFH-DA staining under 400 mM Mannitol stress.

The O_2_^−^ and H_2_O_2_ levels in the transgenic *Arabidopsis* seedlings under drought stress were detected by DAB and NBT staining (Figures 9A,B). The results indicated that both DAB and NBT staining of transgenic and WT seedlings under normal conditions were light, indicating a lower content of O_2_^−^ and H_2_O_2_. Under 400 mM mannitol treatment, however, the seedlings *abc1k3* and *abc1k6* mutants displayed the deepest staining, followed by WT seedlings, and *TaABC1K3 and TaABC1K6* overexpressed seedlings had the lightest staining. These results indicated that simulated drought stress caused a large accumulation of ROS in the seedlings of *abc1k3/6* mutants and WT. The overexpression of *TaABC1K3* and *TaABC1K6* could significantly reduce O_2_^−^ and H_2_O_2_ content and ROS accumulation. DCFH-DA fluorescence staining was further used to measure the changes of ROS content in plants under drought stress (Figure 9C). The results showed that *abc1k3* and *abc1k6* mutants had the highest fluorescence intensity under 400 mM mannitol treatment, followed by WT. *TaABC1K3/6* overexpressed plants showed the lowest fluorescence intensity, indicating that *TaABC1K3* and *TaABC1K6* genes could reduce ROS accumulation and alleviate oxidative stress in plants.

**Figure 10:**
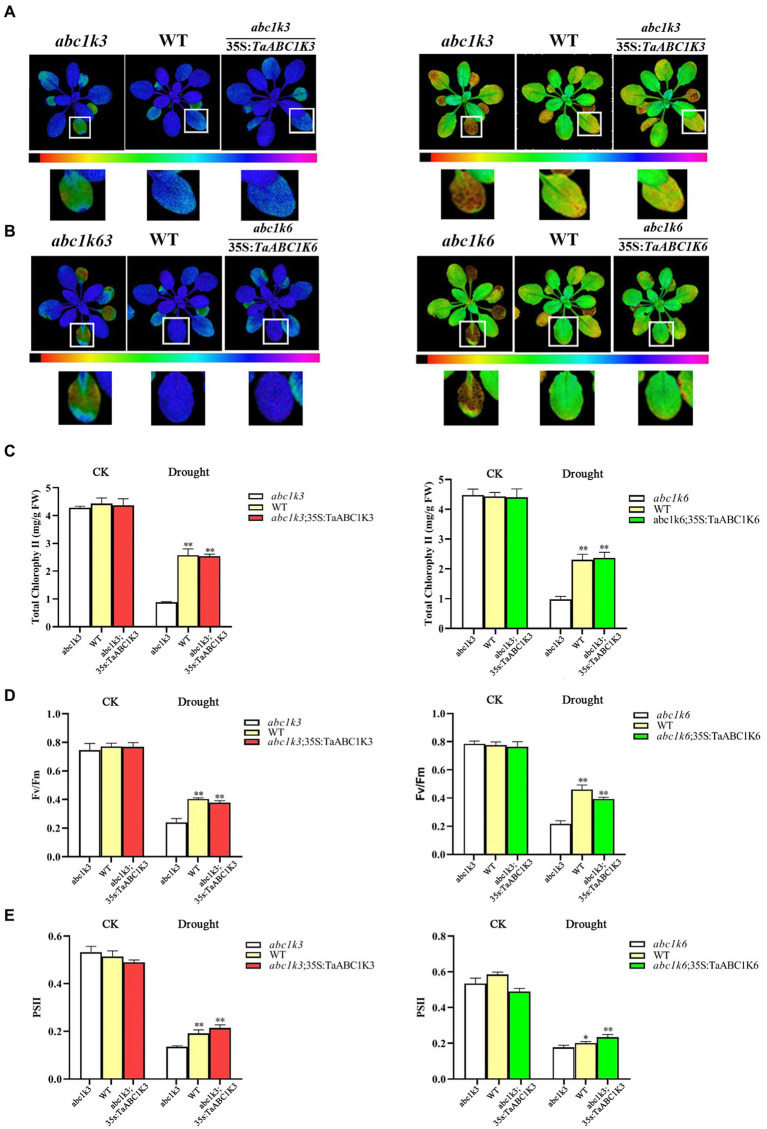
Overexpression of *TaABC1K3* and *TaABC1K6* alleviated the photosynthesis impairment induced by drought stress in *Arabidopsis* mutant *abc1k3* and *abc1k6*. **(A,B)** Chlorophyll fluorescence images showed the efficiency of PSII (FPSII), and the inhibition of PSII quantum yield (lnh) by *abc1k3/6 mutant*, wild type, and completed complementing experimental plants. **(C)** Changes in chlorophyll content of mutant, wild-type, and complemented experimental plants after drought stress treatment. **(D)** Maximum seed yield of each plant (*F_v_/F_m_*) of each plant. **(E)** PSII efficiency value of the plant in chlorophyll fluorescence image. Statistically significant differences between control group and treatment group were calculated by an independent Student’s *t*-tests: *p < 0.05; **p < 0.01.

Chlorophyll content measurement demonstrated that all materials had no significant differences under normal growth conditions (Figures 10A,B). The chloroplast content in all plants was significantly decreased under drought treatment (Figure 10C). In particular, the chloroplast content in *abc1k3* and *abc1k6* was dramatically decreased, respectively, by 80.19 and 79.28% compared to WT. However, *TaABC1K3* and *TaABC1K6* complementation lines showed similar chlorophyll content with WT, indicating that *TaABC1K3/6* genes could enhance chlorophyll synthesis under drought stress. Chlorophyll fluorescence imaging tests also showed similar results, and no significant phenotypic differences were found in the mutant plants, WT plants and the complementation plants under normal conditions. Under drought stress, the maximum photochemical mass yield (*F_v_*/*F_m_*) of PSII in *abc1k3* and *abc1k6* mutant plants under dark adaptation was significantly reduced by 69.01 and 73.84%, respectively (Figure 10D). The photochemical efficiency of PSII (ΦPSII) of *abc1k3* and *abc1k6* was decreased, respectively, by 75.9 and 68.05% while that in the complementary *TaABC1K3* and *TaABC1K6* plants was reduced, respectively, by 56.91 and 53.42% (Figure 10E). These results indicated that *TaABC1K3* and *TaABC1K6* could complement the loss of *Arabidopsis* mutants and enhance chlorophyll synthesis and photochemical efficiency under drought stress.

## Discussion

At present, *ABC1K* genes have been detected in 42 species, including archaea, bacteria, and eukaryotes, which contain three branches in different species (Lundquist et al., 2012a). In allohexaploid wheat, 44 *TaABC1K* genes were also classified into three branches (Figure 1) and evenly distributed on A, B, and D subgenomes (Figure 4). *ABC1K* was considered to be a highly conserved gene family, two functional domains (VAVK-motif and DFG-motif) were found in every *ABC1K* genes in both wheat (Supplementary Figure 2) and rice (Yang et al., 2012c). Positive selection sites were not found among *ABC1K* genes (Supplementary Table 5), indicating that wheat *ABC1K* gene family did not suffer from significant positive selection pressure during the evolution process. It is possible that genes that did not undergo positive selection might have undergone negative or purified selection (Yang, 2007). *ABC1K* genes in rice and *Arabidopsis* mainly experienced purifying selection, and the functions of most *ABC1K* genes tended to be conserved (Gao et al., 2014). However, wheat *ABC1K* genes from different clades showed some structural differences in motif and exon compositions, especially those from clade I (Figure 2), consistent with the previous reports in rice (Yang et al., 2012c) and maize (Gao et al., 2010). Additionally, the 3D structures of TaABC1K proteins predicted by AlphaFold2 also displayed differences in α-helix, β-sheet, and random coil compositions (Figure 3; Supplementary Table 3). These results imply that wheat the functional differentiation of *ABC1K* genes might happen during the evolutionary process. We also found that much more type II function divergence sites were present between clade I and clade II, implying that the differentiation in physicochemical properties played a leading role during the evolutionary process (Zhu et al., 2014). Only type I function divergence sites were found between clade II and clade III (Supplementary Table 4), and the changes in the evolutionary rate might be the main factor for the evolution of the two clades (Han et al., 2019; Liu et al., 2020).

Wheat *ABC1K* genes such as *TaABC1K3* and *TaABC1K6* have abundant *cis*-elements related to environmental stress, including ABRE, MBS, and G-box, etc. (Supplementary Figure 3), which could play important roles in defending various abiotic stresses. Drought stress causes ROS accumulation in plants, and as signal molecules, ROS also participates in various biotic and abiotic stress responses and played an important role in plant growth and development (Suzuki et al., 2011; Marino et al., 2012). Thus, increased ROS accumulation under drought stress could induce the upregulation of *TaABC1K* genes in the leaves and shoots (Supplementary Figure 4; Figures 5B,C), consistent with the previous reports (Wang et al., 2011; Wu et al., 2020). At the same time, the overexpression of *TaABC1K3* and *TaABC1K6* in yeast and *Arabidopsis* significantly promoted drought tolerance (Figures 6, 7). On the other hand, the absence of *Arabidopsis TaABC1K3* and *TaABC1K6* genes led to susceptible to drought while the complementation experiment of *TaABC1K3* and *TaABC1K6* could recover the drought resistance of *Arabidopsis* plants by reducing ROS accumulation caused by drought (Figure 10). *ABC1K10a* mutant in *Arabidopsis* also caused more susceptible to salt stress (Qin et al., 2020), and the double mutations of *Arabidopsis ABC1K AtSIA1* and *AtOSA1* were influenced by oxidative stress even in a normal condition and the SOD level in plants was significantly upregulated (Manara et al., 2014). Abiotic stresses could also produce oxidative damage to photosynthetic proteins and pigments. PSII (*F_v_*/*F_m_*) and Chl content are important indicators of photochemical efficiency, which can reflect physiological aging (Franke and Schreiber, 2007; Lim et al., 2007; Osakabe et al., 2010). The maximal quantum yield of PSII photochemistry (*F_v_*/*F_m_*) of wheat under 3 days osmotic stress was significantly lower than normal conditions. Compared to other stresses, wheat plants under 3 days osmotic stress had the most obvious decline in gas-exchange parameter (Wu et al., 2020). *TaABC1K* overexpressed plants under drought stress showed higher water retention ability and higher *F_v_*/*F_m_* than WT (Wang et al., 2011). In this study, *Arabidopsis abc1k3* and *abc1k6* mutations under drought stress significantly influenced chlorophyll content and photosynthetic efficiency in PSII, while *TaABC1K3* and *TaABC1K6* could complement for the loss of *ABC1Ks* in *Arabidopsis* and maintain the function of chlorophyll synthesis in plants (Figure 10). These results confirmed that *TaABC1K3* and *TaABC1K6* genes could maintain the stability of plant photosynthesis.

Abiotic stresses generally produce excessive production of ROS in plants, leading to damages to proteins, lipids, carbohydrates, and DNA, eventually leading to oxidative stress (Meijer et al., 2019). Non-enzymatic antioxidants such as ascorbic acid (ASC), glutathione (GSH), α-tocopherol, plastoquinone, and carotenoids could detoxify ROS and protect plants from abiotic stress (Bisby et al., 1999; Kruk et al., 2005; Kruk and Trebst, 2008). Studies found that ABC1K could phosphorylate tocopherol cyclase VTE1 *in vitro*, which might protect VTE1 from degradation (Martinis et al., 2013, 2014), and VTE1 was a key enzyme in vitamin E synthesis and recycling (Kobayashi and Della Penna, 2008). As the hydrolysis product of vitamin E, a single α-tocopherol molecule could neutralize up to 220 ^1^O_2_ molecules *in vitro* before being degraded (Gill and Tuteja, 2010). Therefore, ROS in chloroplast could be effectively decreased.

Drought can cause disequilibrium between light capture and its utilization, which reduces the rate of photosynthesis in leaves. This imbalance would induce excess light energy dissipated in the plant photosynthetic system (Mansur and Luiz, 2015). The excess energy absorbed by chlorophyll in the PSII antennae complex would further cause the conversion of singlet chlorophyll to deleterious triplet chlorophyll (^3^Chl*; Pospíšil, 2016). The reaction of ^3^Chl* and O_2_ could produce highly oxidizing ^1^O_2_ (Edreva, 2005). Carotenoids and lutein can scavenge ^1^O_2_ to inhibit oxidative damage and quench ^3^Chl* to prevent the formation of ^1^O_2_, thus protecting the photosynthetic apparatus (Mozzo et al., 2008; Mansur and Luiz, 2015). Zeaxanthin cycleoxidase (ZEP) serves as an important enzyme to synthesize carotenoids in carotenoid metabolism and is involved in the reversible conversion of zeaxanthin to violaxanthin within the xanthophyll cycle (Yuan et al., 2015). The study also found that ZEP was centrally located in the gene expression network closely related to ABC1K, and ABC1K proteins might regulate ZEP activity (Lundquist et al., 2012b). Thus, TaABC1K might coexpressed with ZEP to participate in the regulation of the xanthophyll cycle, and the increasement of carotenoids could scavenge ^1^O_2_ and quench ^3^Chl* to stabilize plant photosynthesis (Lundquist et al., 2012b).

**Figure 11:**
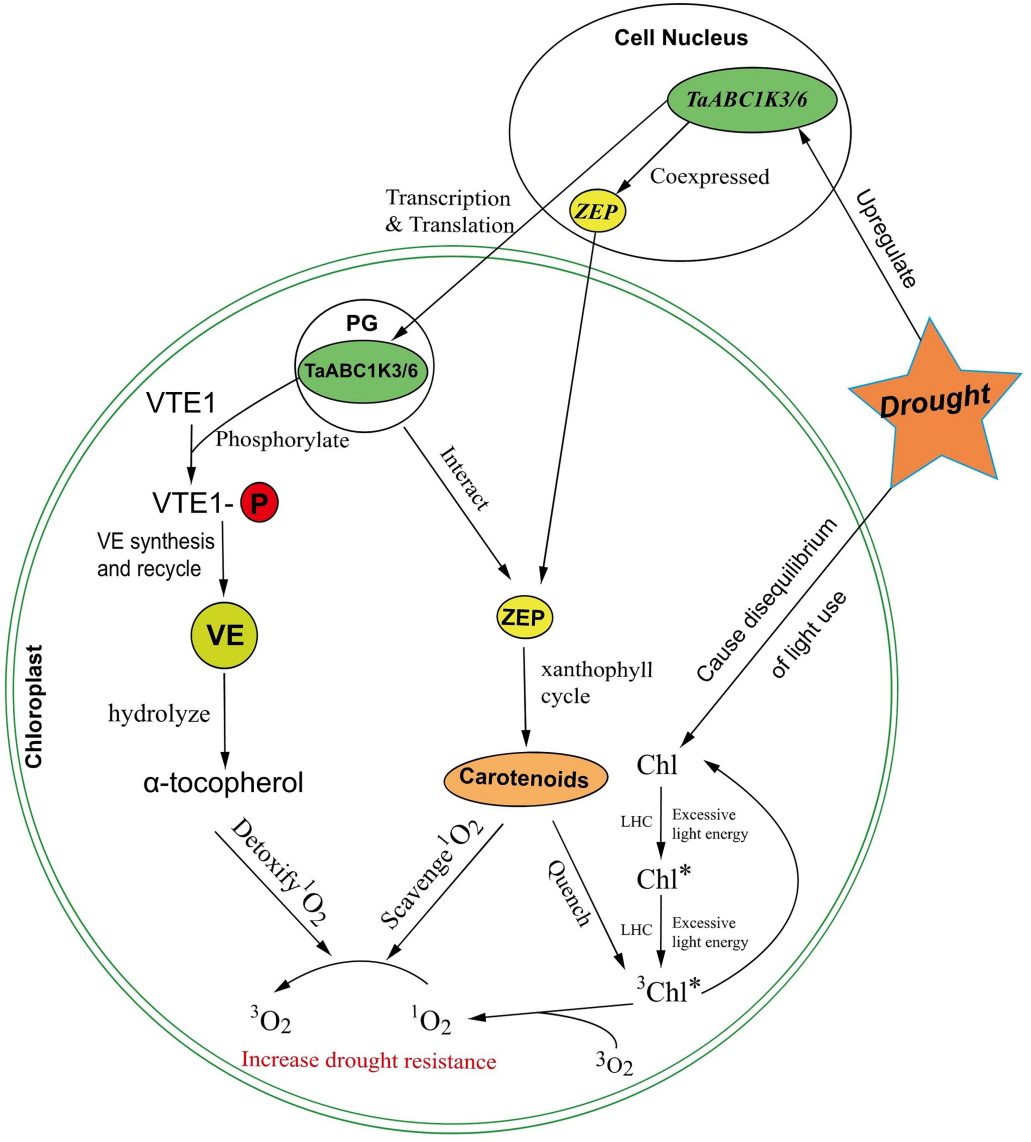
A putative regulated network of *TaABC1K3* and *TaABC1K6* in response to abiotic stress in wheat chloroplast. Symbols: PG, plastoglobuli; LHC, light-harvesting complex; ZEP, zeaxanthin epoxidase; VTE1, tocopherol cyclase; VE, vitamin E.

Based on the results of this work as well as the previous studies, we proposed a putative responsive network of *TaABC1Ks* in wheat chloroplast under drought stress (Figure 11). When subjected to drought stress, the disequilibrium of light use produced excessive light energy to induce the production of ^3^Chl*. The reaction of ^3^Chl* and O_2_ could produce ROS ^1^O_2_. The increased ROS accumulation triggered the upregulated expression of *TaABC1K3* and *TaABC1K6* in the leaves. Carotenoid synthesis was enhanced by TaABC1K3 and TaABC1K6 coexpressing and interacting with ZEP in the chloroplast, which could quench ^3^Chl to normal Chl and scavenge ^1^O_2_. Meanwhile, TaABC1K3 and TaABC1K6 could phosphorylate VTE1 and activate the production of α-tocopherol to detoxify ^1^O_2_. This could significantly decrease ROS accumulation under drought stress. Therefore, *TaABC1K3* and *TaABC1K6* could maintain the normal function of chloroplast by regulating the endogenous oxidation balance of chloroplasts to respond drought stress.

## Conclusion

Genome-wide analysis identified 44 wheat *ABC1K* family genes that contained typical ABC1K kinase domain and three (I–III) clades. Each clade generally had similar structural features, but differences in the number of motifs and exons in *TaABC1K* genes as well as the content of α-helix, random coil and β-sheet in TaABC1K proteins from different clades were present. More II type functional divergence sites were found between clade I and clade III and no positive selection sites were found. *TaABC1K* genes possessed abundant *cis*-acting elements in the upstream promoter regions, including light responsive elements, development-related elements, hormone-responsive elements, and environmental stress-related elements. *TaABC1Ks* generally displayed a high expression in plant leaves and response to drought stress. Overexpression of *TaABC1K3* and *TaABC1K6* in yeast and *Arabidopsis* significantly improved drought tolerance. Furthermore, *TaABC1K3* and *TaABC1K6* could, respectively, complement the function of *Arabidopsis abc1k3* and *abc1k6* mutants and alleviate photosynthesis damage caused by drought stress. Our results provided new insights into the structure, evolution, and function characteristics of wheat *ABC1K* genes.

## Data availability statement

The datasets presented in this study can be found in online repositories. The names of the repository/repositories and accession number(s) can be found in the article/Supplementary material.

## Author contributions

XG, RZ, and HS performed most of the experiments and data analysis. JL and WD performed confocal microscope observation and gene expression analysis. YH and YY designed the experiments and edited the manuscript. All authors contributed to the article and approved the submitted version.

## Funding

This research was financially supported by the grant from the National Natural Science Foundation of China (31971931).

## Conflict of interest

The authors declare that the research was conducted in the absence of any commercial or financial relationships that could be construed as a potential conflict of interest.

## Publisher’s note

All claims expressed in this article are solely those of the authors and do not necessarily represent those of their affiliated organizations, or those of the publisher, the editors and the reviewers. Any product that may be evaluated in this article, or claim that may be made by its manufacturer, is not guaranteed or endorsed by the publisher.

## Footnotes

^1^ https://phytozome-next.jgi.doe.gov/

^2^ http://plants.ensembl.Org/

^3^ http://pfam.xfam.org

^4^ https://web.expasy.org/compute_pi/

^5^ http://www.ebi.ac.uk/Tools/msa/muscle/

^6^ http://gsds.cbi.pku.edu.cn/

^7^ http://meme-suite.org/tools/meme

^8^ http://bioinformatics.psb.ugent.be/-webtools/plantcare/html/

^9^ http://www.wheat-expression.com/

## Supplementary material

SUPPLEMENTARY FIGURE 1 The Bayesian phylogenetic tree of wheat ABC1K gene family.

SUPPLEMENTARY FIGURE 2 Conserved amino acid conservation sequences in the ABC1 domain of the *ABC1K* gene family in wheat. VAVK and DFG motifs are marked.

SUPPLEMENTARY FIGURE 3 The *cis*-acting element analysis in the *TaABC1K* gene promoters. **(A)** Distribution of cis-acting elements in each member of wheat *ABC1K* gene family. **(B)** Average number of *cis*-acting elements contained in subfamily members.

SUPPLEMENTARY FIGURE 4 Expression profile of *TaABC1K* genes in wheat different tissues and developmental stages **(A)** and abiotic stresses **(B)**.

SUPPLEMENTARY FIGURE 5 Identification of *abc1k3/6* mutant, *TaABC1K3/6* overexpressed plants and complement plants. **(A)** Using *Arabidopsis* genomic DNA as modle, chimeric primers were designed for PCR identification of T3 generation *TaABC1K3/6* overexpressed plants and complement plants, Lines 1–15 are overexpressed plant samples (complement plants), Line 16 is wild type negative control, and Line17 is plasmid positive controls. **(B)** The *Arabidopsis* mutant purchased from the Salk site was T-DNA insertion mutation, the *abc1k3* mutant was SALK_128696, and the *abc1k6* mutant was SALK_057147.

## Abbreviations

ABRE: Abscisic acid response element
ARE: Cis-acting regulatory element
ASC: Ascorbate
CAT: Catalase
DAB: 3,3′-Diaminobenzidine
DCFH-DA, 2,7-Dichlorodihydrofluorescein diacetate
DPA: Day post-anthesis
GFP: Green fluorescent protein
GSH: Glutathione
LTR: Long terminal repeat
NBT: Nitrotetrazolium blue chloride
ROS: Reactive oxygen species
RT-qPCR: Quantitative real-time polymerase chain reaction
SOD: Superoxide dismutase
ZEP: Zeaxanthin cycleoxidase
