# Supplementary figures and images for: Genome-wide identification of wheat ABC1K gene family and functional dissection of TaABC1K3 and TaABC1K6 involved in drought tolerance
Source: Front Plant Sci. 2022 Aug 29;13:991171. doi: 10.3389/fpls.2022.991171 (PMC9465391; doi:10.3389/fpls.2022.991171)

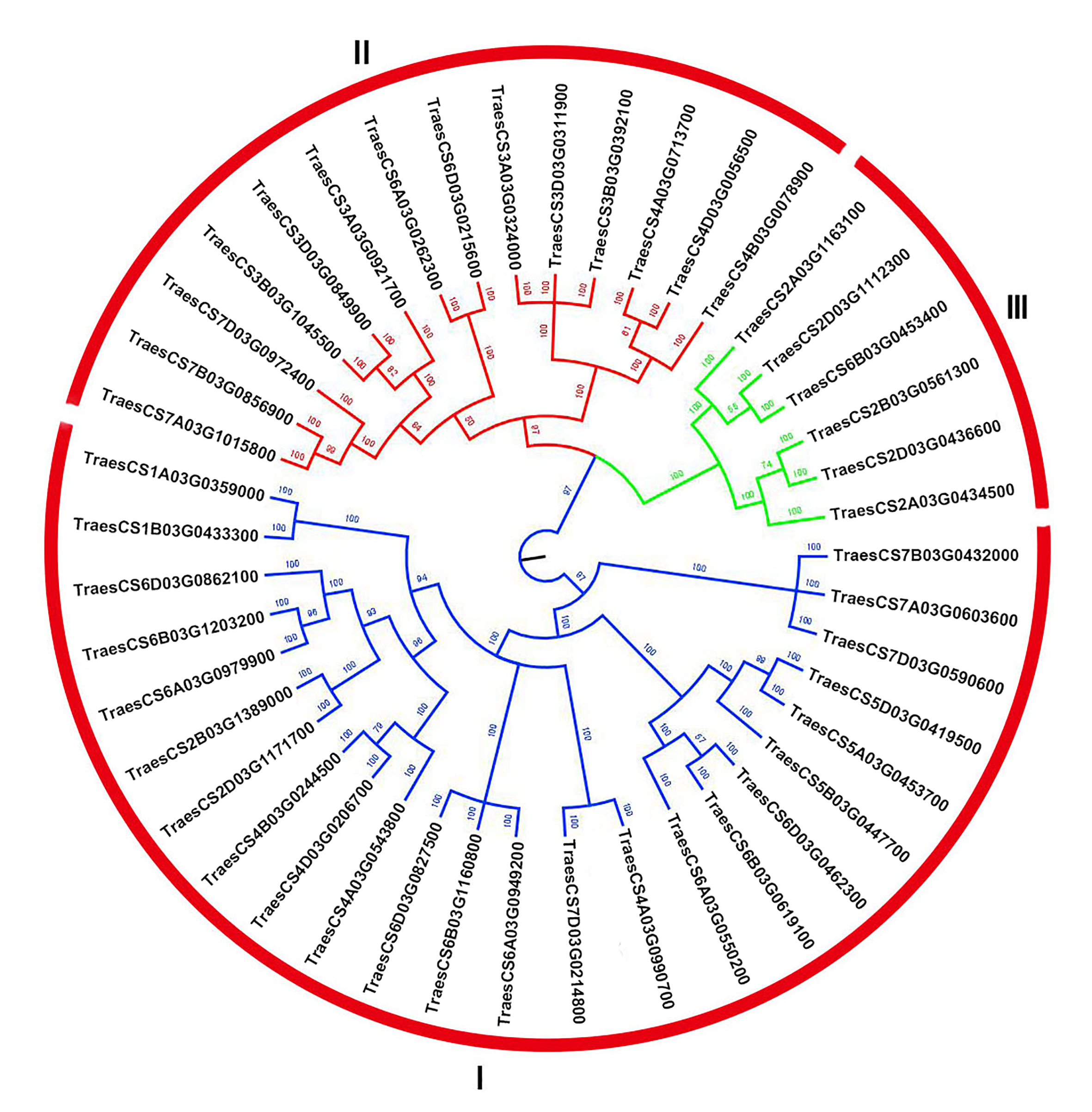

Supplement: SUPPLEMENTARY FIGURE 1 — The Bayesian phylogenetic tree of wheat ABC1K gene family. [file Image_1.JPEG]

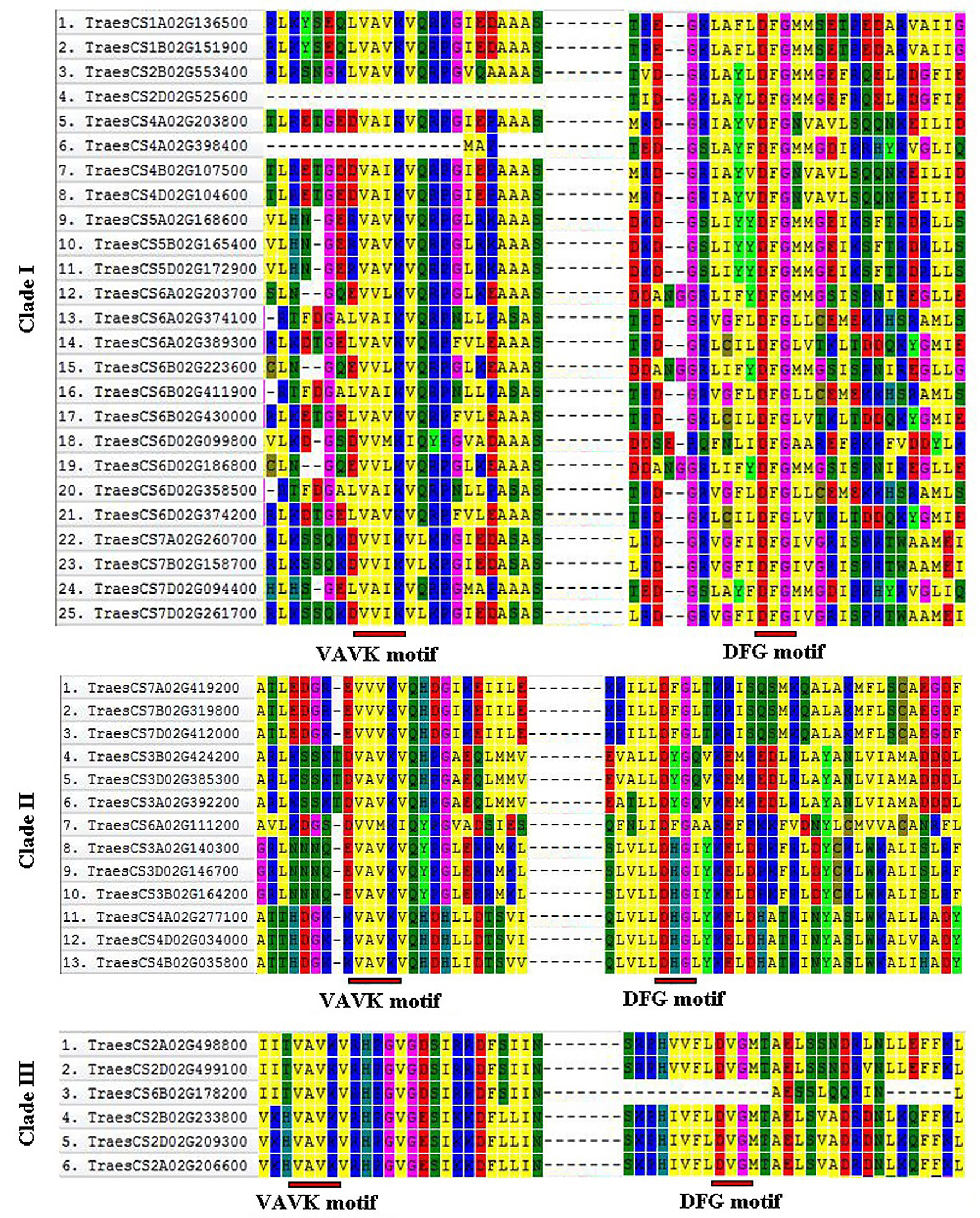

Supplement: SUPPLEMENTARY FIGURE 2 — Conserved amino acid conservation sequences in the ABC1 domain of the ABC1K gene family in wheat. VAVK and DFG motifs are marked. [file Image_2.JPEG]

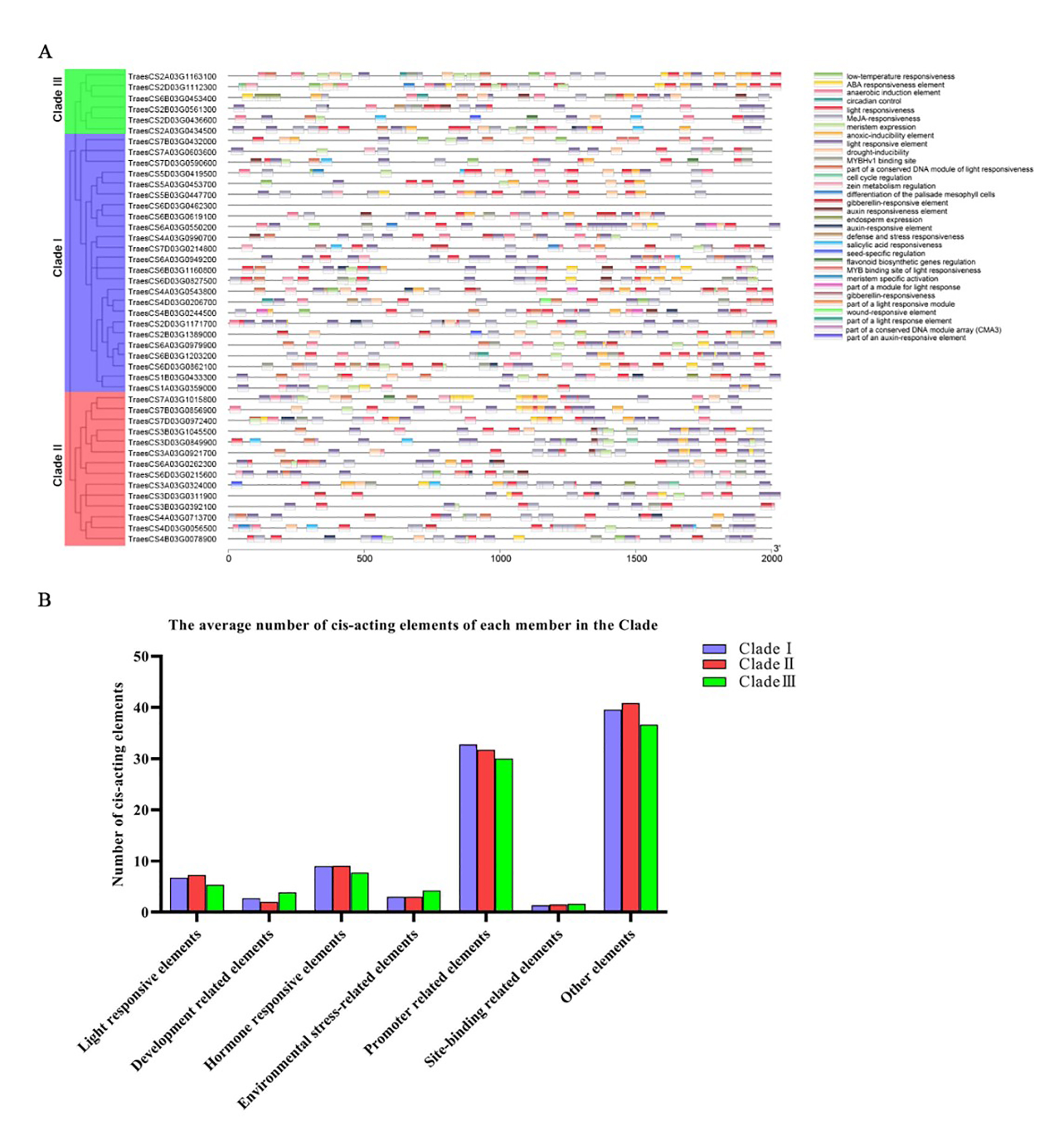

Supplement: SUPPLEMENTARY FIGURE 3 — The cis-acting element analysis in the TaABC1K gene promoters. (A) Distribution of cis-acting elements in each member of wheat ABC1K gene family. (B) Average number of cis-acting elements contained in subfamily members. [file Image_3.JPEG]

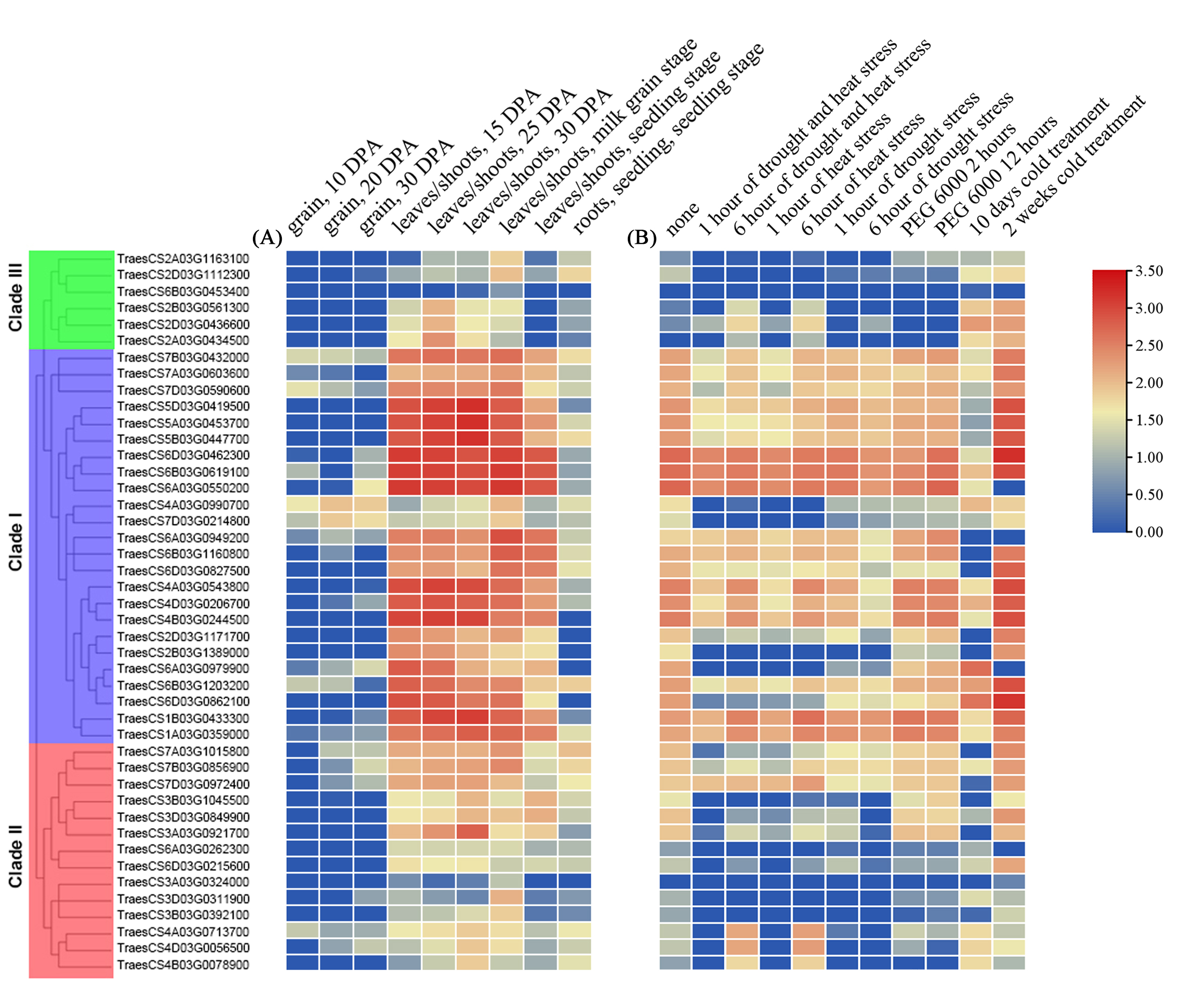

Supplement: SUPPLEMENTARY FIGURE 4 — Expression profile of TaABC1K genes in wheat different tissues and developmental stages (A) and abiotic stresses (B). [file Image_4.JPEG]

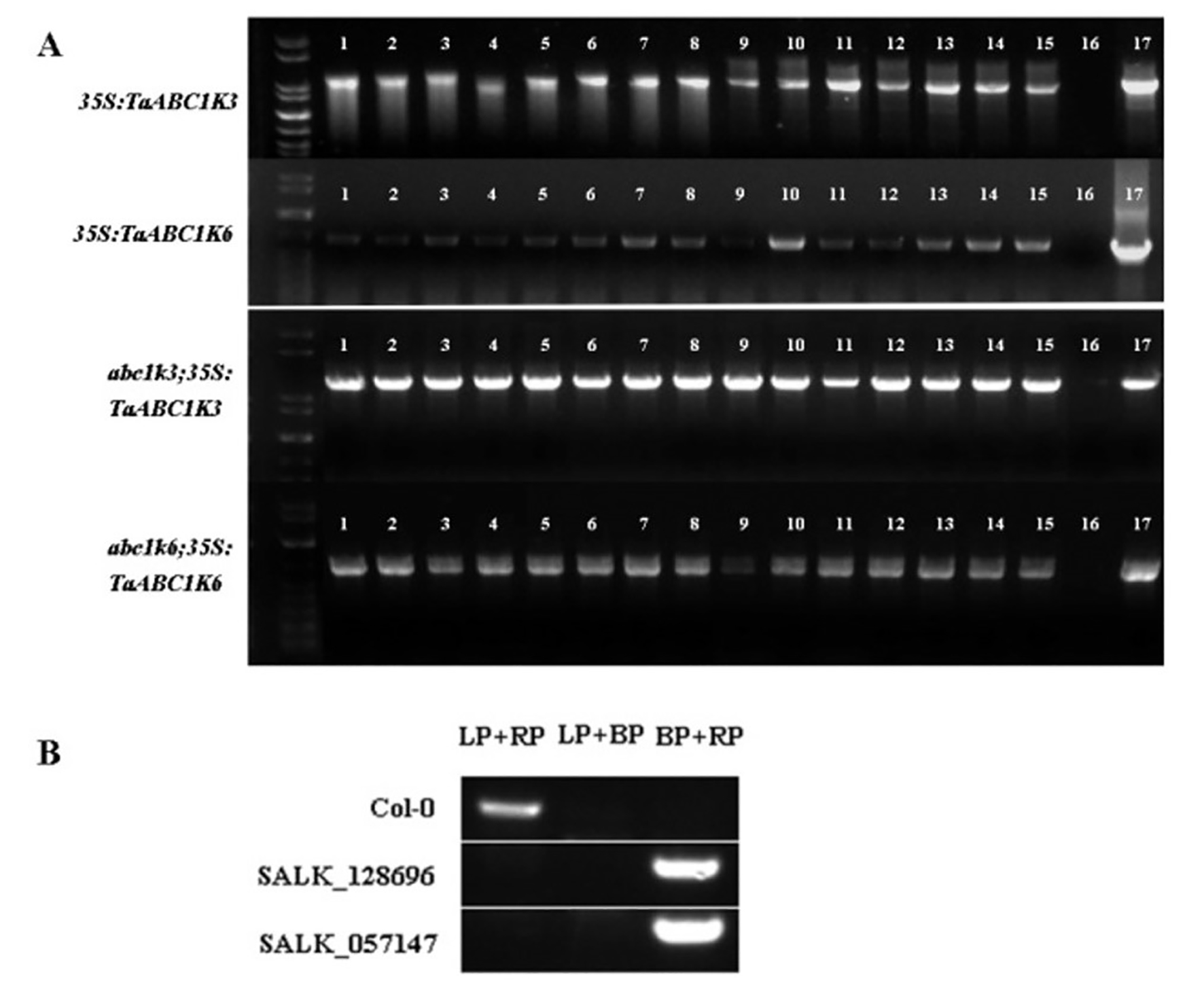

Supplement: SUPPLEMENTARY FIGURE 5 — Identification of abc1k3/6 mutant, TaABC1K3/6 overexpressed plants and complement plants. (A) Using Arabidopsis genomic DNA as modle, chimeric primers were designed for PCR identification of T3 generation TaABC1K3/6 overexpressed plants and complement plants, Lines 1–15 are overexpressed plant samples (complement plants), Line 16 is wild type negative control, and Line17 is plasmid positive controls. (B) The Arabidopsis mutant purchased from the Salk site was T-DNA insertion mutation, the abc1k3 mutant was SALK_128696, and the abc1k6 mutant was SALK_057147. [file Image_5.JPEG]
