# Supplementary material for: Genome-wide identification of wheat ABC1K gene family and functional dissection of TaABC1K3 and TaABC1K6 involved in drought tolerance
Source: Front Plant Sci. 2022 Aug 29;13:991171. doi: 10.3389/fpls.2022.991171 (PMC9465391; doi:10.3389/fpls.2022.991171)
Supplement: Supplementary file 6 [file Table_1.PDF]

Supplementary Table 1. Gene IDs of the *ABC1K* gene family members from eight plant species.

Spicies	Gene ID	Spicies	Gene ID	Spicies	Gene ID
Triticum Aestivum	TraesCS1B02G151900	***Selaginella moellendorffii***	EFJ24335	***Arabidopsis thaliana***	AT4G31390
	TraesCS4A02G203800		EFJ20869		AT5G24970
	TraesCS4A02G398400		EFJ19874		AT1G79600
	TraesCS4D02G104600		EFJ18556		AT2G39190
	TraesCS5D02G172900		EFJ17140		AT1G71810
	TraesCS6A02G374100		EFJ15382		AT3G24190
	TraesCS6B02G411900		EFJ13381		AT3G07700
	TraesCS6D02G186800		EFJ11901		AT5G64940
	TraesCS6D02G358500		EFJ08947		AT5G05200
	TraesCS7A02G099800		EFJ08750		AT1G11390
	TraesCS7A02G419200		EFJ07399		AT1G61640
	TraesCS7B02G319800		EFJ05783		AT5G24810
	TraesCS7D02G094400		EFJ37815		AT4G24810
	TraesCS7D02G412000		EFJ35464		AT5G50330
	TraesCS3A02G140300		EFJ32376		AT4G01660
	TraesCS3D02G146700		EFJ06435		AT1G65950
	TraesCS2A02G498800		EFJ36502		AT2G40090
	TraesCS2B02G233800		EFJ34697	***Physcomitrella patens***	Pp3c10_18310
	TraesCS2D02G209300		EFJ32035		Pp3c15_19180
	TraesCS2D02G499100		EFJ30045		Pp3c17_21120
	TraesCS3B02G164200		EFJ29802		Pp3c18_17830
	TraesCS6B02G178200		EFJ08989		Pp3c1_33470
	TraesCS3B02G424200		EFJ09480		Pp3c21_15420
	TraesCS2A02G206600		EFJ33839		Pp3c21_5070
	TraesCS6A02G111200		EFJ35582		Pp3c24_7050
	TraesCS6D02G099800		EFJ17307		Pp3c5_15400
	TraesCS2D02G525600		EFJ24320		Pp3c6_29720
	TraesCS5A02G168600		EFJ34851		Pp3c9_4120
	TraesCS5B02G165400		EFJ24965		Pp3c5_19100
	TraesCS6A02G203700		EFJ35897		Pp3c13_11180
	TraesCS6A02G389300		EFJ35602		Pp3c6_24460
	TraesCS6B02G430000		EFJ31016		Pp3c15_9220
	TraesCS6D02G374200		EFJ31207		Pp3c13_6150
	TraesCS7B02G158700		EFJ33842		Pp3c22_5080
	TraesCS3A02G392200		EFJ21688	***Populus trichocarpa***	PNS90760
	TraesCS2B02G553400		EFJ21685		PNS92084
	TraesCS3D02G385300		EFJ12241		PNT05351
	TraesCS4A02G277100		EFJ25852		PNT18045

	TraesCS4B02G035800	***Chlamydomonas reinhardtii***	CHLRE_01g 031900		PNT27773
	TraesCS4D02G034000		CHLRE_03g 158500		PNT34086
	TraesCS1A02G136500		CHLRE_04g 215400		PNT35587
	TraesCS4B02G107500		CHLRE_05g 246552		PNT46984
	TraesCS6B02G223600		CHLRE_06g 269801		PNT52778
	TraesCS7A02G260700		CHLRE_07g 325727		PNT48099
	TraesCS7D02G261700		CHLRE_07g 347980		PNS91925
Brachypodium distachyon	KQJ84875		CHLRE_08g 369150		PNT34107
	KQJ85093		CHLRE_09g 407801		PNT40053
	KQJ88241		CHLRE_10g 430800		PNT01229
	KQJ88938		CHLRE_13g 565260		PNT11726
	KQK00012		CHLRE_13g 570350		PNT39343
	KQK01246		CHLRE_13g 581850		PNT23175
	KQK01348		CHLRE_10g 430800		PNT50413
	KQK06851		CHLRE_03g 150600	***Oryza sativa Japonica Group***	Os01t0904200
	KQK07675		CHLRE_06g 307100		Os02t0575500
	KQK11074				Os02t0805800
	KQK12297				Os03t0698350
	KQK15786				Os04t0640500
	KQK16395				Os04t0660200
	KQK17398				Os06t0701300
	KQK13676				Os07t0227800
	KQK04157				Os07t0558000
	PNT62058				Os09t0250700

PNT75193		Os11t0549686
		Os11t0216300
