# Supplementary material for: Genome-wide identification of wheat ABC1K gene family and functional dissection of TaABC1K3 and TaABC1K6 involved in drought tolerance
Source: Front Plant Sci. 2022 Aug 29;13:991171. doi: 10.3389/fpls.2022.991171 (PMC9465391; doi:10.3389/fpls.2022.991171)
Supplement: Supplementary file 7 [file Table_2.PDF]

Supplementary Table 2. Nomenclature and characteristics of *TaABC1K* family genes in *Triticum aestivum* L.

Gene name	Genomics length	Protein length	PI	Mw (Da)	Locations on chromosome
TraesCS1B03G0433300	1610	507aa	5.56	56.2	1B: 239,776,698-239,779,164
TraesCS4A03G0543800	2686	754aa	6.72	84.01	4A: 494,552,371-494,558,815
TraesCS4A03G0990700	1425	474aa	6.1	53.89	4A: 673,443,226-673,448,245
TraesCS4D03G0206700	2560	752aa	5.95	83.91	4D: 82,824,481-82,830,774
TraesCS5D03G0419500	2685	717aa	9.38	80.39	5D: 270,217,814-270,226,961
TraesCS6A03G0949200	2865	954aa	9.45	105.88	6A: 596,905,198-596,912,689
TraesCS6B03G1160800	3151	944aa	9.52	104.61	6B: 685,345,197-685,351,349
TraesCS6D03G0462300	2670	754aa	9.09	85.01	6D: 249,439,707-249,447,151
TraesCS6D03G0827500	2835	944aa	9.51	104.81	6D: 451,177,969-451,183,882
TraesCS7A03G1015800	3137	941aa	6.31	103.67	7A: 610,679,007-610,686,259
TraesCS7B03G0856900	3373	941aa	6.22	103.59	7B: 569,935,604-569,942,478
TraesCS7D03G0214800	2364	708aa	9.01	79.64	7D: 56,941,349-56,948,560
TraesCS7D03G0972400	2787	734aa	5.42	83.22	7D: 530,657,588-530,664,137
TraesCS3A03G0324000	2113	536aa	9.47	60.74	3A: 119,473,835-119,483,574
TraesCS3D03G0311900	2055	536aa	9.34	60.61	3D: 110,411,920-110,420,456
TraesCS2A03G1163100	2736	630aa	9.57	70.68	2A: 728,772,087-728,777,387
TraesCS2B03G0561300	1914	637aa	9.95	72.27	2B: 232,347,194-232,351,404
TraesCS2D03G0436600	2838	637aa	9.94	72.22	2D: 164,991,106-164,996,972
TraesCS2D03G1112300	2801	649aa	9.65	72.61	2D: 594,188,176-594,193,020
TraesCS3B03G0392100	2166	536aa	9.37	60.49	3B: 161,646,618-161,655,279
TraesCS6B03G0453400	1701	380aa	9.68	42.71	6B: 197,736,109-197,739,251
TraesCS3B03G1045500	1588	373aa	7.56	42.56	3B: 661,826,513-661,832,583
TraesCS2A03G0434500	2576	637aa	9.97	72.29	2A: 185,725,023-185,730,351
TraesCS6A03G0262300	2183	607aa	6.03	66.31	6A: 80,686,000-80,691,297
TraesCS6D03G0215600	2234	607aa	6.32	66.41	6D: 62,885,487-62,890,801
TraesCS2D03G1171700	1650	454aa	5.63	50.93	2D: 613,435,143-613,445,580
TraesCS5A03G0453700	2611	717aa	9.38	80.46	5A: 359,039,536-359,049,291
TraesCS5B03G0447700	1964	578aa	9.28	65.02	5B: 306,442,984-306,451,235
TraesCS6A03G0550200	2656	753aa	9	84.76	6A: 349,520,541-349,527,818
TraesCS6A03G0979900	2692	782aa	5.37	86.17	6A: 604,883,222-604,887,720
TraesCS6B03G1203200	3232	785aa	5.44	86.37	6B: 697,752,877-697,758,222
TraesCS6D03G0862100	2896	787aa	5.44	86.62	6D: 458,758,356-458,763,122
TraesCS7B03G0432000	1556	430aa	5.01	48.53	7B: 215,358,241-215,365,454
TraesCS3A03G0921700	2205	477aa	7.56	54.47	3A: 639,701,294-639,711,710
TraesCS2B03G1389000	2508	709aa	6.21	79.21	2B: 748,973,486-748,983,247
TraesCS3D03G0849900	2211	477aa	8.34	54.54	3D: 500,542,623-500,555,571
TraesCS4A03G0713700	2004	532aa	7.68	60.38	4A: 585,094,385-585,099,780
TraesCS4B03G0078900	2026	530aa	7.72	60.24	4B: 25,952,007-25,957,035

TraesCS4D03G0056500	2082	530aa	7.34	60.22	4D: 15,518,460-15,523,943
TraesCS1A03G0359000	2177	601aa	5.2	67.28	1A: 217,610,380-217,613,638
TraesCS4B03G0244500	2567	676aa	5.47	75.55	4B: 117,187,482-117,194,214
TraesCS6B03G0619100	2507	753aa	9.03	84.81	6B: 337,217,569-337,224,625
TraesCS7A03G0603600	2112	543aa	5.95	60.72	7A: 253,800,957-253,808,049
TraesCS7D03G0590600	1962	548aa	6.21	61.18	7D: 237,843,277-237,857,047
