# Supplementary material for: Genome-wide identification of wheat ABC1K gene family and functional dissection of TaABC1K3 and TaABC1K6 involved in drought tolerance
Source: Front Plant Sci. 2022 Aug 29;13:991171. doi: 10.3389/fpls.2022.991171 (PMC9465391; doi:10.3389/fpls.2022.991171)
Supplement: Supplementary file 8 [file Table_3.PDF]

Supplementary Table 3. Percentage of α-helix, random coil and β-sheet of nine ABC1K proteins from different clades.

Clade	Gene name	α-helix (%)		Random coil (%)		β-sheet (%)	
Clade I	TraesCS1B03G0433300	7.5	7.2 ± 0.8^{b}	91.7	92.0 ± 0.4^{b}	0.8	0.8 ± 0.1^{b}
	TraesCS6A03G0979900	6.9		92.4		0.7	
	TraesCS7A03G0603600	7.1		92.0		0.9	
Clade II	TraesCS3D03G0311900	8.2	8.0 ± 0.4^{a}	90.7	90.9 ± 0.3^{c}	1.1	1.1 ± 0.2^{a}
	TraesCS3B03G1045500	7.5		91.2		1.3	
	TraesCS4A03G0713700	8.3		90.7		1.0	
Clade III	TraesCS2D03G0436600	5.8	6.2 ± 0.5^{c}	93.6	93.0 ± 0.6^{a}	0.6	0.7 ± 0.1^{b}
	TraesCS2A03G1163100	6.7		92.5		0.8	
	TraesCS2B03G0561300	6.3		93.0		0.7	

Note: $p<0.05$ was used as significant differences.
