# Supplementary material for: Genome-wide identification of wheat ABC1K gene family and functional dissection of TaABC1K3 and TaABC1K6 involved in drought tolerance
Source: Front Plant Sci. 2022 Aug 29;13:991171. doi: 10.3389/fpls.2022.991171 (PMC9465391; doi:10.3389/fpls.2022.991171)
Supplement: Supplementary file 9 [file Table_4.PDF]

Supplementary Table 4. Functional divergence between clades of the *TaABC1K* gene family.

Group 1	Group 2	Type 1			Type 2	
		θI ± s.e.	LRT	Sites with Qk>0.8	θII ± s.e.	Sites with Qk>0.8
Clade I	Clade II	0.184±0.139	3.835	None	-0.355±1.327	None
Clade I	Clade III	0.399±0.112	11.143	331V, 365Y, 336Q 331G	-0.009±0.706	361Q, 331G, 299F, 296A, 370A, 374P, 354G, 320S, 355I, 333K, 364E, 360R, 321A, 280A, 357C, 284F, 311V, 351V, 285Q, 332V, 273V, 313D, 281R, 291Q, 339A, 310L, 317D, 338A, 348L, 365Y, 282R
Clade II	Clade III	0.070±0.118	4.184	331V	-0.731±0.584	None

Note: θI and θII, the coefficients of type I and type II functional divergence; LRT, likelihood ratio statistic; Qk, posterior probability
