# Supplementary material for: Genome-wide identification of wheat ABC1K gene family and functional dissection of TaABC1K3 and TaABC1K6 involved in drought tolerance
Source: Front Plant Sci. 2022 Aug 29;13:991171. doi: 10.3389/fpls.2022.991171 (PMC9465391; doi:10.3389/fpls.2022.991171)
Supplement: Supplementary file 10 [file Table_5.PDF]

Supplementary Table 5. Positive selection analysis of *TaABC1K* gene family.

Models	nP	InL[a]	2△InL[b]	Positively selected site
M0 (one-ratio)	89	-5706.994512		none
M3 (discrete)	93	-5579.449898	255.09	none
M7 (beta)	90	-5581.948147		none
M8 (beta & ω)	92	-5581.948621	0.000948	none

Note: a, log likelihood; b, twice the log-likelihood
