# Supplementary material for: Genome-wide identification of wheat ABC1K gene family and functional dissection of TaABC1K3 and TaABC1K6 involved in drought tolerance
Source: Front Plant Sci. 2022 Aug 29;13:991171. doi: 10.3389/fpls.2022.991171 (PMC9465391; doi:10.3389/fpls.2022.991171)
Supplement: Supplementary file 11 [file Table_6.PDF]

Supplementary Table 6. Coevolution sites of *TaABC1K* gene family.

Group	Coevolution sites	Group	Coevolution sites
1	6&7	10	127&507
2	6&127	11	228&232
3	6&340	12	340&507
4	6&503	13	382&383
5	6&507	14	455&456
6	27&28	15	466&467
7	67&68	16	503&504
8	78&79	17	503&507
9	127&340	18	507&508

Note: All sites were located on the reference sequence TraesCS1B03G0433300 based in the multiple alignment result.
