# Supplementary material for: Genome-wide identification of wheat ABC1K gene family and functional dissection of TaABC1K3 and TaABC1K6 involved in drought tolerance
Source: Front Plant Sci. 2022 Aug 29;13:991171. doi: 10.3389/fpls.2022.991171 (PMC9465391; doi:10.3389/fpls.2022.991171)
Supplement: Supplementary file 12 [file Table_7.PDF]

Supplementary Table 7. Primers of target genes used for gene clone and vector construction

Primer name	Sequence (5'-3)
163-TaABCIK3-F	TATCTCTAGAGGATCCATGGCGGCATGTACGCCAGCGACGG
163-TaABCIK3-R	TGCTCACCATGGATCCATCAGAGCATCTCTTAAAATTAGGA
163-TaABCIK6-F	TATCTCTAGAGGATCCATGGAGGCCGCCACGGCGCCGCACC
163-TaABCIK3-R	TGCTCACCATGGATCCCAAAAATGATTCCCGGATCACCCGT
1302-TaABC1K3-F	GGGGACTCTTGACCATGGTGATGGCGGCATGTACGC
1302-TaABC1K3-R	GAATTCCTGCAGCACCATGGTAGAAGATCCAGGAACAGGTG
1302-TaABC1K6-F	GGGGACTCITGACCATGGTGATGGAGGCCGCCGCGTCG
1302-TaABC1K6-R	GAATTCCTGCAGCACCATGGTCAAAAATGATTCCCGGAT
TraesCS1B03G0433300-F	ACCTCTGCCCGACTGAGTAT
TraesCS1B03G0433300-R	AACTTGCTGCAGCTATCGGT
TraesCS6A03G0979900-F	CCATCCAGTCCACCAGGTTC
TraesCS6A03G0979900-R	AGTCGCATGGTGACGTCTTT

*Underlines represent homologous arms used for homologous recombination of vector construction
